# Customizing the Therapeutic Response of Signaling Networks to Promote Antitumor Responses by Drug Combinations

**DOI:** 10.3389/fonc.2014.00013

**Published:** 2014-02-05

**Authors:** Alexey Goltsov, Simon P. Langdon, Gregory Goltsov, David J. Harrison, James Bown

**Affiliations:** ^1^Centre for Research in Informatics and Systems Pathology (CRISP), University of Abertay Dundee, Dundee, UK; ^2^Division of Pathology, Institute of Genetics and Molecular Medicine, Western General Hospital, University of Edinburgh, Edinburgh, UK; ^3^Touch Surgery, London, UK; ^4^School of Medicine, University of St Andrews, St Andrews, UK

**Keywords:** anti-cancer combination therapy, cancer drug resistance, pathway engineering, signaling networks, pertuzumab, PI3K/PTEN/AKT

## Abstract

Drug resistance, *de novo* and acquired, pervades cellular signaling networks (SNs) from one signaling motif to another as a result of cancer progression and/or drug intervention. This resistance is one of the key determinants of efficacy in targeted anti-cancer drug therapy. Although poorly understood, drug resistance is already being addressed in combination therapy by selecting drug targets where SN sensitivity increases due to combination components or as a result of *de novo* or acquired mutations. Additionally, successive drug combinations have shown low resistance potential. To promote a rational, systematic development of combination therapies, it is necessary to establish the underlying mechanisms that drive the advantages of combination therapies, and design methods to determine drug targets for combination regimens. Based on a joint systems analysis of cellular SN response and its sensitivity to drug action and oncogenic mutations, we describe an *in silico* method to analyze the targets of drug combinations. Our method explores mechanisms of sensitizing the SN through a combination of two drugs targeting vertical signaling pathways. We propose a paradigm of SN response customization by one drug to both maximize the effect of another drug in combination and promote a robust therapeutic response against oncogenic mutations. The method was applied to customize the response of the ErbB/PI3K/PTEN/AKT pathway by combination of drugs targeting HER2 receptors and proteins in the down-stream pathway. The results of a computational experiment showed that the modification of the SN response from hyperbolic to smooth sigmoid response by manipulation of two drugs in combination leads to greater robustness in therapeutic response against oncogenic mutations determining cancer heterogeneity. The application of this method in drug combination co-development suggests a combined evaluation of inhibition effects together with the capability of drug combinations to suppress resistance mechanisms before they become clinically manifest.

## Introduction

Anti-cancer combination therapies are an increasingly promising way to better treat patients, offering an increase in efficacy of drugs, and a means to overcome/avoid resistance to targeted drug therapy. An evidence base of successes of multidrug strategies in anti-cancer therapy is growing yearly, but typically the mechanism of synergy is not entirely understood. To promote a more directed and systematic development of combination therapies, it is necessary to clearly elucidate the underlying mechanisms that drive the advantages of drug combination effect. Exploration of these mechanisms can impact significantly on the determination and validation of advanced combination of targets in novel drug development strategies such as drug combination co-development (1) and drugdiagnostics co-development (2). With respect to a drug combination co-development strategy, novel compounds should be developed in a combinatorial context and not independently in order to gain those proven integrative benefits with respect to tumor growth inhibition, drug resistance, and toxicity. In the drug–diagnostic co-development model, the drug and the diagnostic assay are developed in conjunction. Companion diagnostic (CDx) assays as well as clinical trial strategies in the case of drug combination therapy should be adapted to multiple biomarkers and different drug combinations (2). Considering the risk of increasing toxicity by the use of drug combination therapy, the US food and drug administration (FDA) released drug co-development guidance, which proposed stringent regulatory recommendations for the use of combination strategies (1, 3). According to these recommendations, there should exist a strong biological rationale for the use of the combinations and proven significant advantages over the use of the drugs as individual agents.

Rational determination and validation of advanced targets for drug combination therapy are the main challenges for these strategies. The inherent complexities of signaling networks (SNs), including crosstalk logic and redundancy in their topology (4, 5) limit the relevance of genotype-based screening to identify and validate optimal drug combination targets among several hundred possible targets. In the face of these challenges, we need more sophisticated methods to design effective combination therapies (1).

Systems biology and computational bioinformatics may offer a key role in advancing drug combination co-development. These technologies are being developed to support rationalized screening of effective drug combinations, and to formulate quantitative criteria for drug combination target validation and specific properties of each drug in a combination context and in the face of different mutational statuses of different cancers (6–9). Such developments are motivated by the potential to reduce the size, duration, and cost of clinical trials as well as to accelerate regulatory approval. Our own studies to support drug combination co-development are described here. Our method is based on analysis of the response of a cellular SN to drug intervention, and its modification by drug combination to achieve high efficacy and reduce drug resistance potential. The approach developed here is founded on the two most attractive advantages of drug combination well established recently: (i) a drug combination suppresses *de novo* or acquired resistance to one of the drugs in the combination – a resistance suppression effect; and (ii) one drug in a combination sensitizes therapeutic response to a second drug – a sensitizing effect.

Resistance suppression effects arising from drug combination are used widely in clinics now, by manipulating pathway dynamics or by slowing down the cancer evolution. Some examples of pathway manipulations are summarized in Ref. (1) targeting different domains of the same protein with two drugs [for example, trastuzumab combined with pertuzumab for human epidermal growth factor receptor, HER2, in breast (10) and ovarian (11, 12) cancers]; horizontally targeting parallel signaling pathways [for example, combined MEK and PI3K/AKT inhibition (13, 14)], and vertically targeting up- and down-stream pathways in one SN [for example, targeting BRAF and MEK, HER2, and PI3K, PI3K/AKT and mTOR (15–17)]. An evolutionary approach (adaptive therapy) to treat drug resistance has been suggested, with the aim of lowering the cancer cell’s capacity to evolve resistance mechanisms through adaptive combination therapy in order to hinder the emergence of resistance types (18). Beyond these approaches, the combination of chemotherapy and molecular targeted therapy demonstrates a complementary mechanism of efficacy and may suggest a route for next-generation cancer therapy (10, 19, 20). For example, a clinical trial of HER2-positive breast cancer progression following HER2 inhibitor trastuzumab therapy showed that trastuzumab in combination with chemotherapeutic agent (capecitabine) was more effective than capecitabine alone. It would seem that trastuzumab sensitizes cell response to other drugs despite the fact that cells are refractory to trastuzumab and that there is therapeutic benefit to continue trastuzumab therapy in combination with other drugs beyond progression (21).

The sensitizing effects of drug combinations are well established in combined therapies targeting different pathways, such as the PI3K/PTEN/AKT and MAPK networks (13, 14); EGFR, DNA-damaging apoptotic signaling pathways (22) as well as in drug combinations inhibiting different nodes in the same pathway: PI3K/PTEN/AKT/mTOR (23–25), Ras/RAF/MEK/ERK (26, 27). However, there are longer-term implications arising from drug combinations since they may lead to different mutations and negatively impact resistance potential. For example, comparison of pre- and post-trastuzumab treatment of tissue samples in metastatic HER2-amplified breast cancer from patients progressing on trastuzumab allowed determination of mutations in *PIK3CA* and *PTEN* arising during treatment (4). A significant reduction, or full loss, of PTEN expression was observed in trastuzumab refractory metastatic tumors compared to the cohort untreated by trastuzumab. It was suggested that PTEN expression reduction was acquired as a result of trastuzumab therapy, leading itself to trastuzumab resistance (4). In contrast, treatment by everolimus, an mTOR inhibitor, did not increase mutational load in pre/post treatment samples of renal carcinomas (28). Moreover, this phenomenon is context specific: for example, abiraterone alone and given in combination with other drugs to control side effects contributes to resistance by activating mutations in the hormone receptor genes; however, a different combination including abiraterone was reported to delay drug resistance (29).

These examples highlight the complexities of combination drug therapy design, including the change in sensitivity to mutation and resistance potential through combination action, and the attending challenges associated with validation. To help overcome these complexities and challenges, we propose a method for the *in vitro*/*in vivo*/*in silico* validation of the targets of drug combinations and quantitative estimation of the perturbations induced by drugs in SNs. Our method is based on the study of drug response characteristics of (i) a SN and its modification by different oncogenic mutations and drug action; and (ii) perturbation of SN sensitivity to drug action induced by mutations. Using sensitivity analysis (SA) of this signaling response, we show that inhibition of various targets by the first drug significantly sensitizes SN response to both compensating mutations leading to resistance and the second drug in combination. We present a scheme for customizing the SN response through drug combination to enhance the robustness of the therapeutic response due to weakening of the drug-sensitizing effect to compensation mutations. To demonstrate the method and to study in detail the drug/mutation modification of SN response, we used the kinetic model of drug combination targeting HER2 receptors and vertical PI3K/PTEN/AKT signaling pathway (30–32).

## Materials and Methods

**Figure 1:**
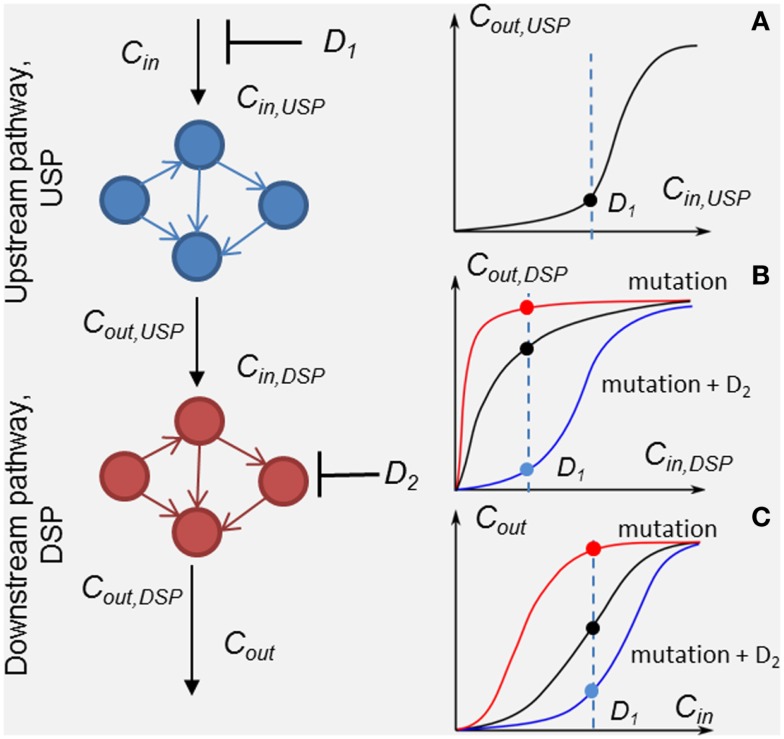
**Scheme of the method of customizing cellular signaling response by drug combination targeting upstream (drug *D*_1_) and down-stream pathway (drug *D*_2_)**. Plot sketches A, B, and C show responses, *R*_USP_ = *C*_out,USP_ (*C*_in,USP_, *D*_1_), *R*_DSP_ = *C*_out,DSP_ (*C*_in,DSP_, *D*_2_), and the whole SN *R*_SN_ = *C*_out_ (*C*_in_, *D*_1_, *D*_2_), respectively. Examples of the responses of unmodified DSP (black line), modified by a mutation in the DSP (red lines) and modified by the second drug *D*_2_ (blue lines) in the presence of the mutation. Input signal *C*_in_ inhibited by drug *D*_1_ is shown by the dashed lines.

The method is based on the analysis of input/output (I/O) responses of a cellular SN, *R*_SN_ = *C*_out_ (*C*_in_, *P*_SN_, *D, t*), which describes the relation between output signal *C*_out_ and input signal *C*_in_ (see scheme in Figure 1). *R*_SN_ depends on molecular parameters, *P*_SN_, of the SN components: specifically, kinetic parameters of the proteins/receptors, *K* = *k*_1_, …, *k_n_* (dissociation constants, catalytic rates), and their expression levels, *E* = *e*_1_, …, *e_m_*. *R*_SN_ also depends on the concentrations of drugs, *D*, which inhibit proteins in the SN. Typically, *R*_SN_ is measured in experimental systems as the dose dependence of output signal on drug/ligand concentration at a defined time, *t*, following ligand/drug application in a cellular assay. The experimental dose dependence, *R*_SN_ = *C*_out_ (*C*_in_, *P*_SN_, *D, t*), on drug concentration allows the definition of the IC_50_ of drug *D*_1_, which strongly depends on the target of drug *D*_2_, the concentration of this second drug, and the mutational status of the SN determined by parameters, *P*_SN_.

To analyze in detail the combination effect of two drugs at different mutations of the SN, we decompose the SN into up- and down-stream pathways that are the targets of drugs *D*_1_ and *D*_2_, respectively (see Figure 1). The response, *R*_SN_, of the SN can be represented through the response *R*_USP_ of upstream pathway (USP) and the response, *R*_DSP_ of down-stream pathway (DSP) in the form, *R*_SN_ = *R*_DSP_(*R*_USP_), where *R*_USP_ = *C*_out,UPS_(*C*_in_*,P*_USP_, *D*_1_, *t*) and *R*_DSP_ = *C*_out,DSP_(*C*_in,DSP_,*P*_DSP_, *D*_2_, *t*). In this formulation, we took into account equivalences of I/O signals: *C*_in,DSP_ = *C*_out,USP_ and *C*_out_DSP_ = *C*_out_ (see Figure 1). Theoretically, the I/O responses, *R*_SN_, *R*_USP_, and *R*_DSP_, are readily calculated from the model of the SN. Experimentally, *R*_DSP_ can be extracted from two experimental dose dependencies: dose dependence of the whole SN, *R*_SN_, and the receptor activation/inhibition dose dependence on activator/inhibitor concentration, *R*_USP_, and represented in the form *R*_DSP_ = *C*_out_(*R*_USP_,*P*_DSP_,*D*_2_,*t*).

Input/output response *R*_DSP_ determines the output signal amplitude of the DSP depending on input signals, which is controlled by the first drug *D*_1_, the molecular parameters, *P*_DSP_, and the action of the second drug *D*_2_. Analysis of *R*_DSP_ allows us to study the effect of mutations, i.e., changes in kinetic properties and expression levels of proteins, *P*_DSP_, on the change in *R*_DSP_ and how these changes determine a transition from drug sensitivity to drug resistance. Plots A, B, and C (black lines) in Figure 1 show arbitrary responses, *R*_USP_, *R*_DSP_, and *R*_SN_, corresponding to sensitivity of the SN to *D*_1_. According to this type of smooth hyperbolic *R*_DSP_, the output signal of the SN changes when the input signal changes under inhibitor *D*_1_ (see black points in plots A, B, and C showing inhibited signal at drug concentration *D*_1_ in Figure 1). The example of I/O response *R*_DSP_ modified by oncogenic mutations is shown in plot B in Figure 1 (red line). This type of switch-like *R*_DSP_ corresponds to resistance of the SN to drug *D*_1_ because the output signal of the SN does not change when the input signal changes under the action of inhibitor *D*_1_ (see red lines and points in plots B and C in Figure 1). Our method provides a means to search for the protein target for drug *D*_2_ to modify the response, *R*_DSP_, in such a way as to reach two complementary effects: first, an increase in inhibition effectiveness of drug *D*_1_ (see, e.g., *R*_DSP_ modified by drug *D*_2_ in plot B, blue line in Figure 1); and second, to ensure robustness of this inhibition effect against different mutations in DSP (see below). One example of such a modified *R*_DSP_ is shown by the blue line in plot B in Figure 1.

To evaluate the robustness of the response *R*_DSP_, we supplemented the analysis of the shape of the response curve with the SA of the DSP to both external and internal perturbations: i.e., changes in input signal and alterations of kinetic parameters and expression level of the proteins involved, *P*_DSP_. We define the relative sensitivity, *S*_DSP_, of the DSP to the input signal as the relative change of output signal *C*_out,DSP_ in response to a relative change in input signal *C*_in,DSP_, which can be written through changes in I/O responses, *R*_USP_ and *R*_DPS_:

(1) $$S_{\text{DSP}}\left(C_{\text{in,DSP}},P_{\text{DSP}},D_{2},t\right)=\frac{\Delta C_{\text{out,DSP}}}{\Delta C_{\text{in,DSP}}}=\frac{\Delta R_{\text{DSP}}∕R_{\text{DSP}}}{\Delta R_{\text{USP}}∕R_{\text{USP}}}.$$

To analyze the sensitivity of the DSP response to different mutations causing changes in protein parameters (phosphorylation rate and expression level), we calculate an absolute value of relative sensitivity to changes in individual parameters *p* of proteins:

(2) $$S_{\text{DSP,p}}\left(C_{\text{in,DSP}},P_{\text{DSP}},D_{2},t\right)=\left|\frac{\Delta R_{\text{DSP}}∕R_{\text{DSP}}}{\Delta p∕p}\right|.$$

We base our local SA on the results of the application of SA to a study of the responses of various cellular signaling pathways to different mutations and drug actions. For the first time, the computational analysis of the change in sensitivity of SN depending on the protein expression level was carried out in the modeling of the apoptosis pathway (33). The SA revealed a sensitivity increase in the response of the apoptotic pathway to overexpression of Bcl-2 protein. It allowed prediction of selectivity of a Bcl-2 inhibitor for tumor cells with Bcl-2 overexpression against healthy cells with a normal level of Bcl-2. SA was first used to analyze the robustness of the MAPK cascade to oncogenic mutations in Ref. (34). The further application of the SA to an *in silico* study of the effects of the most frequent mutations in cancer (EGFR, Ras, BRaf) on the dynamics of MAPK response to receptors activation were analyzed in Refs. (35–39). An application of the SA to a study of SN response to drug action was first studied in Refs. (40, 41).

Sensitivity analysis of the I/O response allows us to: (i) determine the resistance potential of a single drug and drug combination to protein mutations in the DSP; and (ii) determine whether the DSP response to a specific drug combination is robust against various mutations in the DSP and suggest more robust combinations, which enhance the therapeutic effect in the face of mutations.

## Results

### Characterizing signaling network response to different oncogenic mutations

We illustrate this method by application to the analysis of the response of PI3K/PTEN/AKT signaling to a drug combination targeting the HER2 receptor and a protein in the DSP, PI3K, which were established to be promising drug targets in both mono- and combination therapy in different cancers (1, 15, 42). The aim of the analysis of known drugs and targets is to elucidate the mechanism underlying high efficacy of their combination against different oncogenic mutations in cancer (43, 44).

**Figure 2:**
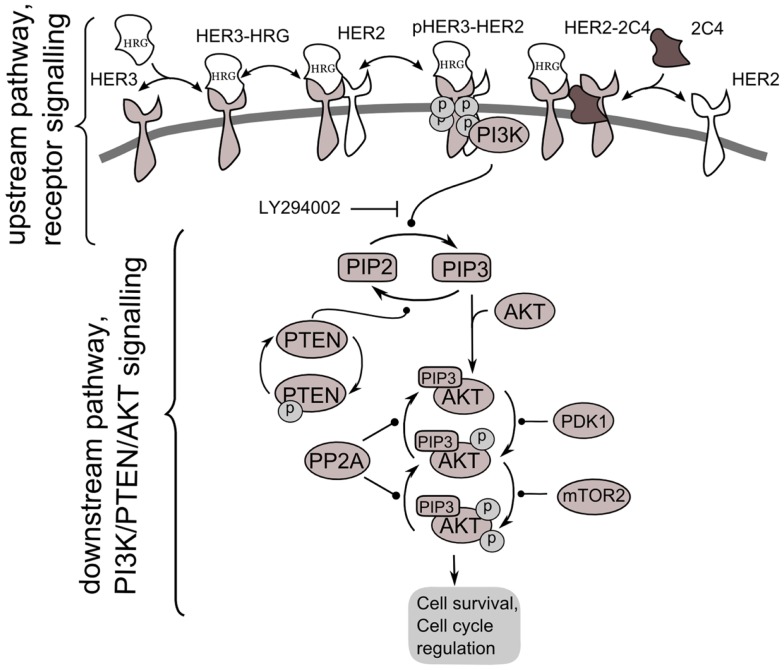
**Scheme of PI3K/PTEN/AKT signaling network activated by heterodimerization of HER2/HER3 receptors induced by heregulin (HRG) and inhibited by pertuzumab, 2C4**. Decomposition of the signaling network to upstream pathway (receptor signaling pathway) and down-stream pathway (PI3K/PTEN/AKT network) is shown.

The analysis is based on the kinetic model of Ras/RAF/MEK/ERK and PI3K/PTEN/AKT signaling developed in Ref. (30). This model describes the response kinetics of the SN to heregulin (HRG)-induced HER3/HER2 receptor heterodimerization and the effect of HER2 inhibitor, pertuzumab (2C4 antibody), on ERK and AKT activation in the human ovarian carcinoma cell line PE04. The scheme of the PI3K/PTEN/AKT SN that corresponds to the SN undertaken here is shown in Figure 2. The ordinary differential equations (ODEs) of the model and set of the model parameters are given in the Supplementary Information. In the model, we neglected other ErbB receptor heterodimers because HER2/HER3 heterodimerization activation was found to be the most mitogenic signal and induces cellular growth in the PE04 cell line (45). The model of the SN including 56 ODEs, 58 reactions, and almost 100 parameters (kinetic constants and protein concentrations) was parameterized by experimental data on the phosphorylation kinetics of HER2, ERK, AKT, and PTEN in the absence and presence of pertuzumab (30). Considering incomplete identifiability of model parameters based on the limited set of experimental data used in model calibration, we validated the model on independent experimental data on the different combination effects of PTEN, PI3K, and HER2 inhibition on the ErbB/PI3K/PTEN/AKT pathway activation (30, 32, 40, 41, 46). Model validation evidenced a good account of sensitivity of the SN to single drug action (pertuzumab) and drug combinations targeting different nodes of the SN (pertuzumab and inhibitors of PI3K, PDK1, and PTEN) (32, 40, 41, 46).

According to our method, we decomposed the ErbB/PI3K/PTEN/AKT network into two sub-networks: an upstream signaling system – the receptor system constituting HER2/HER3 receptor signaling (USP); and a down-stream pathway constituting PI3K/PTEN/AKT pathway (DSP). We used the phospho-heterodimer HER23 signal (*p*HER23) as the output signal of USP and input signal of the DSP (see Figure 2). The I/O response, *R*_USP_, of USP was defined as the dependence of *p*HER23 concentration on the concentration of HER2 inhibitor, 2C4: *R*_HER_ = *p*HER23(HRG, *P*_USP_, 2C4, *t*). The I/O response of DSP, *R*_AKT_, is the dependence of *p*AKT on concentration of phospho-heterodimer HER3/HER2, *p*HER23: *R*_AKT_ = *p*AKT(*p*HER23, *P*_AKT_, *D_2_, t*). The input signal of PI3K/PTEN/AKT is the receptor phosphorylation signal, *p*HER23, which is changed in the calculation by varying the concentrations of the HER2 inhibitor, 2C4, in the physiological region from zero to saturated value, 1 μM (47). The dose dependence, *R*_AKT_, was calculated at time *t* = 30 min after HRG and 2C4 addition that corresponds to saturation of *p*HER23 and *p*AKT signals both in modeling and experiment (30, 32).

**Figure 3:**
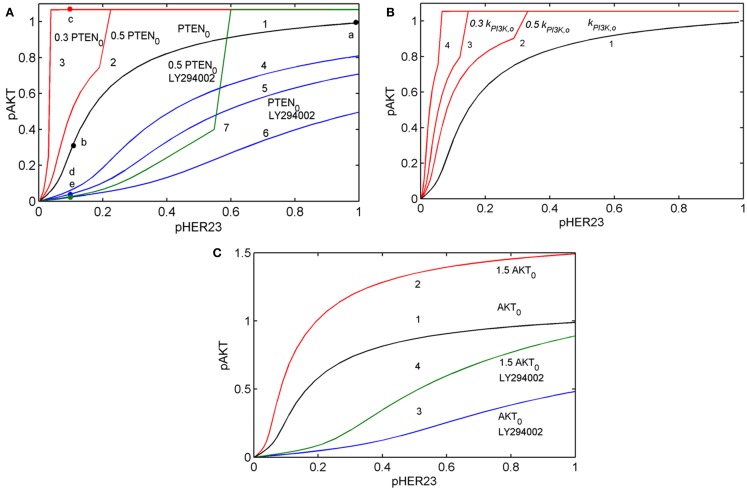
**Input/output response of the PI3K/PTEN/AKT pathway *R*_AKT_ to *p*HER23 input signal and its modification by activation mutations of PTEN, PI3K, and AKT proteins and inhibition of PI3K by LY294002 inhibitor**. **(A)** Modification of *R*_AKT_ by a change of initial concentration of PTEN: response curve at the reference concentration of PTEN_0_ = 40 μM (line 1), 50% PTEN loss (line 2), 70% PTEN loss (line 3). Modification of *R*_AKT_ by PI3K inhibition: 0.3 μM LY294002 (line 4), 0.5 μM LY294002 (line 5), and 1 μM LY294002 (line 6). Modification of *R*_AKT_ by 50% PTEN loss and inhibition of PI3K by 3 μM LY294002 (line 7). Points a, b, c, d, and e correspond to saturated *p*AKT signal (*p*HER23 = 1), its inhibition by 100 nM 2C4 (*p*HER23 = 0.1), resistance to 2C4 at 50% PTEN loss (*p*HER23 = 0.1), combined inhibition by 2C4 and LY294002 in the absence and presence of 50% PTEN loss (*p*HER23 = 0.1), respectively. **(B)** Modification of *R*_AKT_ by a change of PI3K activities, *k*_PI3K_: reference *k*_PI3K,0_ (line 1), 0.5 *k*_PI3K,0_ (line 2), 0.3 *k*_PI3K,0_ (line 3), and 0.1 *k*_PI3K,0_ (line 4). **(C)** Modification of *R*_AKT_ by 50% overexpression of AKT: reference concentration AKT_0_ (line 1) and 1.5 AKT_0_ (line 2). Modification of *R*_AKT_ by 1 μM LY294002 at reference concentration of AKT_0_ (line 3) and 50% overexpression of AKT (line 4).

To show how the I/O response *R*_AKT_ depends on mutations in the PI3K/PTEN/AKT pathway, we compared the theoretical response, *R*_AKT_ = *p*AKT(*p*HER23, *P*_AKT_, *D*_2_, *t*) for the unperturbed DSP (see black line in Figure 3A) with the response *R*_AKT_ modified by oncogenic mutations in the PI3K/PTEN/AKT pathway: PTEN loss (50 and 70%), variation of PI3K activity (Figure 3B), and 50% overexpression of AKT (see red lines in Figures 3A–C).

Comparing the shapes of unmodified and modified responses curves *R*_AKT_, we defined sensitive and resistance modes in the DSP. In sensitive mode, an increase in *p*HER23 signal causes a gradual increase in *p*AKT signal from 0 to its saturated value (black line in Figure 3A). In resistance mode, the *R*_AKT_ curve becomes steeper and transforms to a switch-like response curve at the activation mutations (red lines in Figures 3A,B). The *p*AKT output signal is unresponsive to input *p*HER23 signal controlled by *D*_1_: e.g., *p*AKT signal does not change when *p*HER23 changes in the range from 0.04 to 1 at 70% PTEN loss and activated mutation in PI3K (see red lines 3 and 4 in Figures 3A,B, respectively). In the case of AKT overexpression, *p*AKT signal significantly exceeds the basal activation level of *p*AKT in the range of *p*HER23 signal from 0.04 to 1 (see red line in Figure 3C). This lack of AKT response to input signal at pathway modification by mutations corresponds to the resistance of *p*AKT signal to HER2 inhibition by pertuzumab. Amongst the modifications of response *R*_AKT_ shown in Figures 3A–C, the most pronounced transition from a graded to a steep switch-like shape was observed at PTEN loss. In the model, this effect is due to the post-translational regulation of PTEN activity by its phosphorylation (see Figure 2) leading to the additional loss of PTEN activity (31, 48).

Analysis of *R*_AKT_ shows how receptor inhibition of *p*HER23 signal by *D*_1_ transforms the functioning state of the DSP from its normal function at saturated receptor signal (*p*HER23 = 1) to non-saturated inhibited signal at low *p*HER23 <0.3 (points a and b in Figure 3A, respectively). When the DSP functions in non-saturation mode, the inhibited input signal differs from that in (normal) saturation mode in both a decrease in *p*AKT signal and an increase in sensitivity of the DSP to both input signal *p*HER23 and mutations causing changes in kinetic parameters and expression level of the proteins involved. To study the second effect, we analyzed the behavior of sensitivities of the DSP *S*_DSP_ Eq. 1 and *S*_DSP,p_ Eq. 2 at different internal (mutations) and external (inhibition) modifications of the DSP.

The relative sensitivity of the PI3K/PTEN/AKT pathway is defined as the relative response (change) of the output signal of DSP, Δ*p*AKT, to a relative change in its input signal, Δ*p*HER23:

(3) $$S_{\text{AKT}}\left(p\text{HER}23,P_{\text{AKT}},D_{2},t\right)=\frac{\Delta p\text{AKT}∕p\text{AKT}}{\Delta p\text{HER}23∕p\text{HER}23}.$$

**Figure 4:**
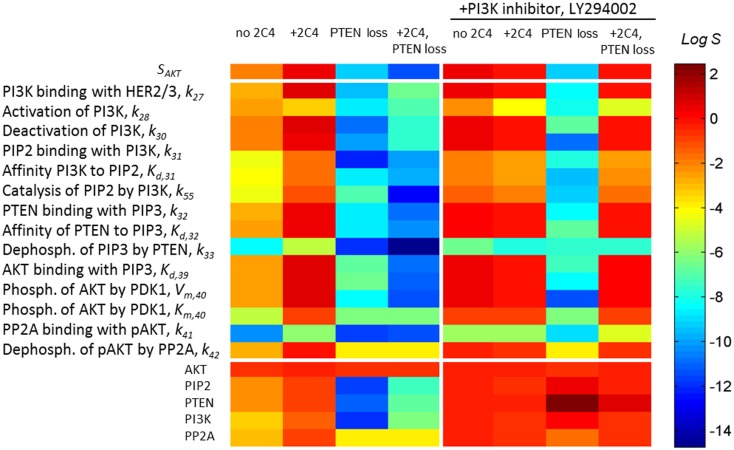
**A change in sensitivities *S*_AKT_ and *S*_AKT,p_ following different perturbations in the PI3K/PTEN/AKT network**. Heatmap of sensitivity *S*_AKT_ to *p*HER23 signal (upper heatmap) and sensitivity *S*_AKT,p_ to kinetic parameters (middle heatmap) and initial protein concentrations (lower heatmap) at normal functioning of the SN (column 1), in the presence of 100 nM 2C4 (column 2), and at 50% PTEN loss in the absence (column 3) and presence of 100 nM 2C4 (column 4). Columns 5–8 show *S*_ATP,p_ as per columns 1–4 in the presence of 3 μM LY294002. The relative change of the parameters in sensitivity analysis was 0.01% (see Eq. 4) and sensitivity values were taken at time *t* = 30 min after HRG and 2C4 addition.

Sensitivity *S*_AKT_ (Eq. 3) was calculated at time *t* = 30 min after HRG and 2C4 addition that corresponds to the saturation of both *p*HER23 and *p*AKT signals. Information on *S*_AKT_ can be obtained based on an analysis of the tangent of the response curve *R*_AKT_: the steeper the response behaviors, the more sensitive the system responses to the external signal. Commonly, sensitivity increases at low input signal and this corresponds in our case to receptor signal inhibition by the first drug (see Figure 3A). At high receptor signal, *S*_AKT_ decreases at saturated signal, *p*HER23 ≅ 1. We represent the theoretical sensitivity *S*_AKT_ (Eq. 3) as the upper heatmap in Figure 4 (columns 1–4) calculated at different perturbations of the DSP: (1) at the activation of DSP (*p*HER23 = 1); (2) at the presence of 100 nM 2C4 (*p*HER23 = 0.1); (3) 50% PTEN loss (*p*HER23 = 1); and (4) 50% PTEN loss with 100 nM 2C4 (*p*HER23 = 0.1). According to the shape change in response curve under these perturbations (see Figure 3A), the sensitivity *S*_AKT_ increases under 2C4 treatment in sensitive mode (compare columns 1 and 2) and decreases at 50% PTEN loss, corresponding to resistance mode (compare columns 1 with 3 and 4).

We also observed that the sensitivity of the DSP *S*_AKT,p_ to protein parameters and expression level increases in the same region of *p*HER23 signal (40). Relative sensitivities *S*_AKT,p_ (Eq. 2) of the PI3K/PTEN/AKT pathway to a specific parameter *p* (including kinetic parameters and expression levels of the signaling proteins) were determined as follows:

(4) $$S_{\text{AKT,p}}\left(p\text{HER}23,P_{\text{AKT}},D_{2},t\right)=\left|\frac{\Delta p\text{AKT}∕p\text{AKT}}{\Delta p∕p}\right|.$$

To show how drug and protein mutations change sensitivity, *S*_AKT,p_ (Eq. 4), we calculated and compared *S*_AKT,p_ for kinetic parameters and initial concentrations of proteins involved in the PI3K/PTEN/AKT network under a normal functioning of the DPS and the mutations considered above. Figure 4 (middle and lower heatmaps, columns 1–4) shows *S*_STS,p_ calculated at: (1) HER23 activation by HRG (*p*HER23 = 1); (2) in the presence of HER2 inhibitor, 2C4, when *p*HER23 signal is 90% inhibited (*p*HER23 = 0.1); (3) at 50% PTEN loss (*p*HER23 = 1); and (4) at 50% PTEN loss in the presence of 2C4 (*p*HER23 = 0.1) (see columns 1–4 in Figure 4, respectively). Analysis of *S*_AKT,p_ at normal signaling in the PI3K/PTEN/AKT network (*p*HER23 = 1) revealed the most sensitive modules within this network (see column 1 in Figure 4). The results obtained in our model are in agreement with the results of local and global sensitivity analyses of other models of the PI3K/PTEN/AKT pathway (41, 49, 50).

We observed a two-to-four orders of magnitude increase in *S*_AKT,p_ in response to changes in the kinetic parameters and initial concentrations of the proteins at 90% inhibition of *p*HER23 signal by 100 nM 2C4 (compare columns 1 and 2 in Figure 4, lower heatmap). This increase correlates with an increase in sensitivity, *S*_AKT_, at an inhibited signal of *p*HER23 (see upper heatmap in Figure 4). The increase in sensitivities *S*_AKT_ and *S*_STS,p_ at low *p*HER23 signals corresponds to the transition of the DSP from functioning in saturated mode (*p*HER23 ≅ 1) to functioning at HER2 inhibition (non-saturation). This correlation in the behavior of *S*_AKT_ and *S*_STS,p_ can be readily understood: sensitivity, *S*_AKT,p_(*p*HER23, *P*_AKT_), to a change in *P*_AKT_ varies in accordance with sensitivity *S*_AKT_ (*pHER2, P*_AKT_) to a change in input signal, *p*HER23:

(5) $$\frac{\Delta S_{\text{AKT}}\left(p\text{HER}23,P_{\text{AKT}}\right)}{\Delta p}=\frac{\Delta S_{\text{AKT,p}}\left(p\text{HER}23,P_{\text{AKT}}\right)}{\Delta p\text{HER}23},$$

where Δ*S*_AKT_ and Δ*S*_AKT,p_ denote sensitivity changes. Equation 5 can be derived from well-known equality of mixed derivatives for continuous function of many variables, turning to infinitesimal increments Δ in Eq. 5 – in our case for I/O response function *p*AKT(*p*HER23, *P*_AKT_) depending on variables *p*HER23 and *P*_AKT_ taken at fixed time *t*.

We applied the results of this analysis to study the change in SN sensitivity to oncogenic mutations in the PI3K/PTEN/AKT pathway. Given an increase in sensitivity *S*_AKT,p_ to all the parameters and expression levels of the proteins of the DSP to external perturbation (+2C4) (see column 2 in Figure 4, lower heatmap), we calculated sensitivity *S*_AKT,p_ at internal perturbations: 50% PTEN loss (column 3 in Figure 4, lower heatmap), variation of PI3K catalytic rate, and 50% overexpression of AKT (data not shown). For all perturbations, we observed the same dramatic fall in sensitivity, *S*_STS,p_, over almost all parameters and this drop in *S*_STS,p_ endows the DSP with insensitivity to any further changes in kinetic parameters of proteins and their abundances. The fall of *S*_STS,p_ correlates with the shape change of the response curve *p*AKT(*p*HER23, *P*_AKT_) (red lines in Figure 3) and the loss of sensitivity *S*_AKT_ to input receptor signal *p*HER23 inhibited by 2C4. In contrast to a sensitive network, this insensitive DSP cannot be sensitized by drug *D*_1_ inhibiting the input signal: the calculation of *S*_AKT,p_ at 100 nM 2C4 showed no change in sensitivity following 50% PTEN loss (see column 4, lower heatmap in Figure 4). Thus, drug *D*_1_ (2C4) acts in very different ways on unmodified and modified DSPs: it sensitizes the unmodified network (sensitive mode), but does not change sensitivity and inhibition level in the mutated DSP. Further *D*_1_, inhibiting the input signal, shifts the DSP to a state with more sensitivity to compensation mutations, which may in turn cause the restoration of the initially activated signal and also lower network sensitivities to other external and internal perturbations.

### Customizing signaling network response through drug combination

Our analysis of the sensitivity of the PI3K/PTEN/AKT pathway showed that inhibition of input signal *p*HER23 sensitizes the response of the DSP to protein perturbations such as a change in protein expression level. Such perturbations can be also exerted by inhibition of protein phosphorylation activity. Here, we use the observed drug-induced sensitizing effect to enhance the action of the second drug *D*_2_ targeting the DSP. Specifically, we considered PI3K inhibition by LY294002 and calculated the response of PI3K/PTEN/AKT pathway, *R*_AKT_ = *p*AKT(*p*HER23, *P*_AKT_, *D*_2_, *t*) to *p*HER23 signal at different concentrations of this inhibitor. Inhibition of PI3K modifies the response curve from hyperbolic to a smooth sigmoid form with high inhibition effect (see blue lines in Figures 3A,C). This modified response has a most pronounced inhibition effect of the second drug *D*_2_ in the region of low *p*HER23 signal (0–0.2) where the sensitizing effect of drug *D*_1_ is maximal (see black lines in Figures 3A,C and column 2 in Figure 4). This observation confirms that the effective and synergistic effect of the HER2 inhibitor (*D*_1_) in combination with PI3K inhibitor (*D*_2_) is due to a sensitizing effect of *D*_1_ at low *p*HER23 signals. The key indicator of the synergistic effect of *D*_1_ and *D*_2_ is a change in the curvature of the response curve from convex to concave at low *p*HER23 signal (0.1–0.3). We assume that modification of the response curve defines the synergistic effect of HER2 and PI3K inhibitor combination (*D*_1_ and *D*_2_). Below, we show that this effect corresponds to a significant decrease of the IC_50_ of pertuzumab in the presence of LY294002 (see Modification of the Dose Response and its Sensitivity by Drug Combination in the Presence of Mutations).

The additional benefit of this transformation of the response curve is an insensitivity of the modified response (smooth sigmoidal shape) to oncogenic mutations in the PI3K/PTEN/AKT pathway at low *p*HER23 signals. To show the acquired robustness of the network in the face of these mutations, we calculated the response, *R*_AKT_ = *p*AKT(*p*HER23, *P*_AKT_), at 50% PTEN loss and AKT overexpression in the presence of 3 μM LY294002 (green lines in Figures 3A,C). As can be seen, PTEN loss and AKT overexpression did not change significantly the inhibited signal *p*AKT in the range of inhibited input signal, *p*HER23 < 0.3 (compare state d and e in Figure 3A and points on lines 3 and 4 at *p*HER23 = 0.1 in Figure 3C, respectively). Thus, in contrast to our results on resistance to 2C4 induced by PTEN loss and AKT overexpression, these abnormalities in the context of PI3K inhibition did not result in resistance to HER2 inhibition by 2C4. Note that the advantages of that drug combination vanish at higher signals *p*HER23 > 0.4 where mutations and protein overexpression significantly increase *p*AKT signal (see lines 6 and 7 in Figure 3A and lines 3 and 4 in Figure 3C). Therefore, the response is more robust than an unmodified hyperbolic one with respect to the activation of mutations in the PI3K/PTEN/AKT pathway at inhibited *p*HER23 signal (*p*HER23 < 0.3).

This advantage of a modified smooth sigmoidal response curve was confirmed by the calculation of sensitivities, *S*_AKT_ and *S*_AKT,p_, carried out in the presence of PI3K inhibitor (columns 5–8 in Figure 4). As can be seen, at this modification, 90% inhibition of *p*HER23 by pertuzumab causes a decrease in sensitivities, *S*_AKT_ and *S*_AKT,p_, in contrast to their increase at the pertuzumab treatment alone (compare columns 2 and 6 in Figure 4). A decrease in sensitivity *S*_AKT_ at low *p*HER23 concentrations (row 1 in column 6 in Figure 4) confers robustness of 90% *p*AKT inhibition in a wider range of inhibited *p*HER23 signal (up to 0.3; see lines 4–6 in Figure 3A and lines 3 and 4 in Figure 3C) than in the case of hyperbolic response in the absence of the second drug. A slight decrease in *S*_AKT,p_ in turn leads to less sensitivity to oncogenic mutations in the PI3K/PTEN/AKT pathway and suppression of drug resistance in contrast to the case of pertuzumab treatment alone. As can been seen from the I/O response curve, the robust inhibition of *p*AKT at low *p*HER23 signal is independent of the oncogenic mutations (mainly 50% PTEN loss and AKT overexpression) (compare points d and e in Figure 3A and points on lines 3 and 4 in Figure 3C at *p*HER23 = 0.1). Note this insensitivity to mutations at this drug combination vanishes at higher input signals *p*HER23 > 0.4, where mutations and protein overexpression significantly increase *p*AKT signal (see, e.g., *p*AKT values at *p*HER23 = 0.6 on lines 6 and 7 in Figure 3A and lines 3 and 4 in Figure 3C). Thus, the combination of HER2 inhibitor with PI3K inhibition endows the DSP with robustness against activation mutations in PI3K/PTEN/AKT pathway only at inhibited *p*HER23 signal in the range up to *p*HER23 = 0.3.

Note that in the model, drug *D*_2_ causes an increase in the sensitivity *S*_AKT,p_ to the protein parameters in comparison with the unperturbed SN that can sensitize the DSP to compensatory mutations (see columns 1 and 5 in Figure 4). This discrepancy may be due to the fact that in the calculation we compared the sensitivity at inhibited (*p*HER23 = 0.1) and saturated (*p*HER23 = 1) *p*HER23 signals (points d and a in Figure 3A, respectively). Here we assumed that the activation growth factor signal (1 nM HRG) is saturated (point a in Figure 3A). However, if we assume ligand concentration to be lower than the saturation level (e.g., 0.6–0.8) (51) and compare the sensitivities at inhibited and this *p*HER23 signal, we find that drug *D*_2_ does not change the sensitivity in comparison with the unperturbed DSP (data not shown).

### Modification of the dose response and its sensitivity by drug combination in the presence of mutations

**Figure 5:**
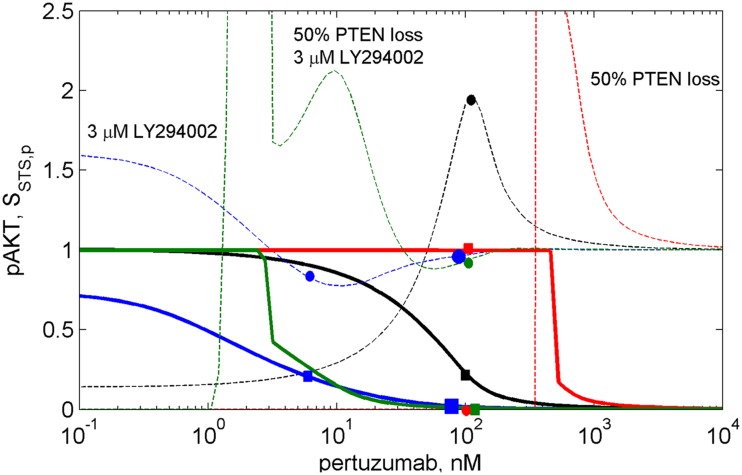
**The dose dependences of *p*AKT inhibition (solid lines) and sensitivity *S*_AKT,k31_(2C4,*p*) of the PI3K/PTEN/AKT network to the rate constant of the reaction of PI3K binding with PIP2, *k*_31_ (dash lines) at different drug combinations**. The dose dependencies of inhibition and sensitivity on pertuzumab concentration (black lines), at 50% PTEN loss (red lines), at 3 μM LY294002 (blue lines), at 50% PTEN loss and 3 μM LY294002 (green lines).

To show how the modifications of I/O response of the AKT subsystem, *R*_AKT_, effect on the dose dependence of the whole ErbB/PI3K/PTEN/AKT network for drug *D*_1_, we calculated *p*AKT and *S*_AKT,p_ dose dependencies for pertuzumab at different perturbations of the PI3K/PTEN/AKT subsystem discussed above (see solid and dashed lines in Figure 5, respectively). Specifically, we calculated sensitivity *S*_AKT,k31_ to the rate constant of the reaction of PI3K binding with PIP2, *k*_31_ (see Figure 2 and model description in Supplementary Information). The calculation for other parameters *p* was shown to effect the same results (as guaranteed by the general behavior of *S*_AKT,p_ for all *p*, according to the discussion of Eq. 5). In the case of an unperturbed DSP, sensitivity *S*_AKT,k31_ increases by approximately 20 times (relative to its level in the absence of pertuzumab) and has a peak at 80% inhibition of *p*AKT (IC_80_ for pertuzumab) (see black circle and square on the dashed and solid black lines at 100 nM pertuzumab in Figure 5, respectively). Key features of the dose dependence *S*_AKT,k31_ calculated with the unperturbed PI3K/AKT module are the presence of maximum in the range of IC_80_, a non-zero limit at high drug concentrations, and a limiting value at low drug concentration, which corresponds to the sensitivity of the signaling system in the absence of the drug (see heatmap in Figure 4). The detailed analysis of these features is given in Supplementary Information (see Figures S1 and S2 in Supplementary Material).

The calculation of the *p*AKT dose dependencies for pertuzumab in the presence of PI3K inhibitor showed the shift of the dose dependence curve to lower drug concentrations and a decrease of IC_50_ for pertuzumab from 30 nM to 1 nM at an increase in LY294002 concentration up to 3 μM (see solid blue line in Figure 5 and Figure S2 in Supplementary Material). In contrast to dose dependence, calculation of relative sensitivity *S*_AKT,k31_ revealed a complex behavior that only moves to low pertuzumab concentrations at increasing LY294002 concentration: the maximum near IC_80_ disappears and sensitivity behavior becomes almost monotonically decreasing with a slight minimum (see Figure S2A in Supplementary Material). We assume that a shape change of *S*_AKT,k31_ at the shift of the dose dependence is the manifestation of transformation of the DSP response curve (Figure 3A). Sensitivity *S*_AKT,k31_ at 3 μM LY294002 does not have maximum at IC_80_ and decreases from its initial value at low 2C4 concentration to approximately its limit value at high pertuzumab concentration (see blue circle and square on the dashed and solid blue lines, respectively at 6 nM pertuzumab in Figure 5). This dose dependence corresponds to the modified response curve (line 6 in Figure 3A). We predict a significant decrease of IC_50_ for pertuzumab in the presence of 3 μM LY294002 due to the synergistic combination of these two drugs and explain this effect by modification of the response of PI3K/PTEN/AKT pathway at low input signals (line 6 in Figure 3A).

Calculation of absolute sensitivity, *S*_AKT,k31_ (Eq. S2.1 in Supplementary Material), showed that it possesses similar features except for a zero limiting value at high drug concentration (see Figure S2B in Supplementary Material). Similarly, at 3 μM LY294002, absolute sensitivity loses its maximum in the range of IC_80_ and monotonically decreases when drug concentration increases. We consider this transformation of sensitivity behavior as the results of modification of the DSP dose response from hyperbolic to smooth sigmoid shape (Figure 3). This transformation, and the range of second drug concentration where it occurs, can be used in optimization of drug composition to inhibit signaling, so avoiding a significant increase in sensitivity of the SNs to mutations. Below we consider one such optimization.

Transformation of the response of the DSP module leads to *p*AKT inhibition by two drugs to exceed 80% in the wide range of LY294002 concentrations (0.3–3 μM) at a significantly lower concentration of pertuzumab (6 nM) in comparison with *p*AKT inhibition by pertuzumab alone, 100 nM (see Figure 5; Figure S2 in Supplementary Material). This allows optimization of drug concentrations with respect to both inhibition of *p*AKT signal and sensitivity of the SN to mutations. Since these two characteristics are interlinked and change correlatively in the same region of the response curve (Figure 3A) and dose dependence (Figure 5), the problem of trade-off between them arises. It is possible to disentangle inhibition and sensitivity characteristics from each other by optimal manipulation of two drugs in the concentration range discussed above. One can establish two optimized conditions of drug combination action with high inhibition and low sensitivity. The first optimum condition is to decrease 2C4 concentration to maintain 80% inhibition of *p*AKT (IC_80_ = 6 nM) with low sensitivity at 2–3 μM LY294002 (see blue square and circle on solid and dashed blue lines respectively in Figure 5 and lines 5 and 6 in Figure S2 in Supplementary Material). The second optimum is full inhibition of *p*AKT with 100 nM pertuzumab and a minimal increase (six times) of sensitivity at 3 μM LY294002 (see large blue square and circle on solid and dashed blue lines respectively in Figure 5).

To demonstrate the sensitivity to resistance transition in response to HER2 inhibition at PTEN loss, we calculated the dose dependences of *p*AKT and *S*_AKT,p_ at 50% PTEN loss (see red solid and dashed lines in Figure 5 and corresponding I/O response of the *p*AKT, line 2 in Figure 3A). PTEN loss leads to a 10-fold shift of *p*AKT dependence to a higher pertuzumab concentration, so causing resistance to HER2 inhibition in the range of physiological pertuzumab concentration (100 nM). In this region of drug concentration, both inhibition of *p*AKT and sensitivity *S*_AKT,p_ are approximately zero (see red square and circle on solid and dashed red lines in Figure 5, respectively). Sensitivity to HER2 inhibition is restored through modification of the network by PI3K inhibitor, LY294002. This modification causes approximately full inhibition of *p*AKT (green square on green solid line in Figure 5) while sensitivity *S*_AKT,p_ (green circle on green dashed line in Figure 5) is at approximately the same level as for normal PTEN concentration.

Joint analysis of the I/O response of the PI3K/PTEN/AKT pathway and *p*AKT dose dependence for pertuzumab (drug *D*_1_) revealed a trade-off between inhibition and sensitivity, which can be formulated as follows. If the concentration of drug *D*_1_ is high (low *p*HER23 signal), *p*AKT inhibition is strong and sensitivity to protein parameters is low: this is of a benefit with respect to signal inhibition and suppression of compensatory mutations. If the concentration of *D*_1_ is low (high *p*HER23 signal), *p*AKT concentration increases while sensitivity decreases, which gives advantages with respect to mutation suppression but leads to a decrease in inhibition effect of the drug, *D*_1_. If the concentration of drug *D*_1_ is in the region of its IC_50_, sensitivity increases which affords the use of the second drug more effectively (drug-sensitizing effect), and a disadvantage with respect to increasing sensitivity of the SN to compensation mutations. This analysis showed the inhibition-sensitivity trade-off decision can be optimized by customizing the I/O response of the SN through manipulation of the concentrations of the two drugs. Optimization of drug concentration allows the separation of the regions with high inhibition and high sensitivity, which overlap each other in single drug treatment.

## Discussion

We have shown that the sensitivity of the SN to drug action is attended by an increase in sensitivity *S*_DSP,p_ of the DSP to the kinetic parameters and expression levels of the proteins involved in this pathway. As a result, the DSP is sensitized by drug action and may be more fragile with respect to mutations, which change protein kinetic properties (catalytic or/and dissociation constants) and their expression level (overexpression or suppression of gene expression). In particular, an increasing sensitivity may result in an adverse effect of inhibitor action since a high sensitivity endows the SN with fragility with respect to mutations that can compensate for the intended inhibitor effect by restoring a high output signal as well as initial low sensitivity of the SN to external perturbations. It follows that increasing SN sensitivity can be one of the causes of resistance potential to drug action. We suggest that this effect should be taken into account at drug target validation, and the drug resistance potential linked with this sensitizing effect should be evaluated in drug combination co-development. We present a method to design drug combination strategies that modify the I/O response of the SN to minimize any drug-sensitizing effect, enhance robustness of drug inhibition effect, and improve drug resistance potential by vertically targeting the SN.

**Figure 6:**
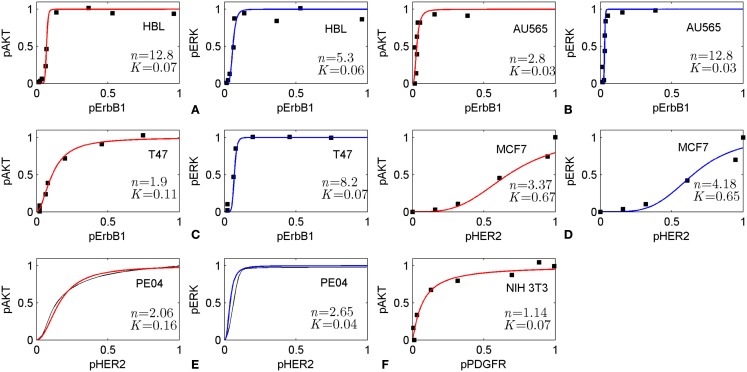
**Input/output responses of PI3K/AKT and MAPK pathways to the activation of growth factor receptors in different cells**. The dependences of *p*AKT and *p*ERK on the activation level of ErbB1 receptors in HBL100 **(A)**, AU565 **(B)**, T47 **(C)** cells; on the level of HER2 receptors in MCF7 **(D)** and PE04 **(E)** cells; and platelet-derived growth factor receptor (PDGF) in NIH 3T3 fibroblasts **(F)**. Data on I/O response were extracted from experimental dose dependences [**(A–C)** (49), **(D)** (54), and **(F)** (55)] and normalized at maximal values of input/output signals. *p*AKT, *p*ERK dose dependencies for PE04 cells **(E)** are theoretical data (40) (black lines). Experimental data were fitted by Hill function: *y* = *x^n^/(x^n^* + *K^n^)* with Hill constant, *n*, and half-maximum constant, *K*, which are given in Figures. Hill function is depicted by red lines for *p*AKT and blue lines for *p*ERK. Experimental data were kindly allowed to be used by PK Sorger **(A–C)**, M Hatakeyama **(D)**, and JM Haugh **(E)**.

The method is based on the modularity approach to analyzing the efficacy of combination therapy developed by Fitzgerald et al. (52). According to this approach, we divide the signaling system into up- and down-stream pathways, which are the targets of the first and second drugs, respectively (see scheme in Figure 1). In this work, we focused on the I/O response characteristics of the down-stream pathways, DSP, which is a signal transduction module in the whole signaling pathway. As shown in Ref. (52), the effect of drug combination significantly depends on the I/O response properties of the receptor systems, and mainly receptor expression level, that shifts the IC_50_ concentration of drug, *D*_1_. To exclude the effects determined by the I/O response properties of the receptor system, we considered only the output signal of the USP which is varied from zero to a saturated level. In this modularity approach, the effect of drug *D*_1_ defines only the input signal for the I/O response curve of the DSP (see Figures 1 and 3A). We expanded this approach developed by Fitzgerald et al. (52) to investigate the modification of the I/O response of the down-stream pathways as a result of external and internal perturbations such as inhibition of signaling proteins and protein mutations. The analysis of the responsiveness of the whole system to the first drug was shown to depend significantly on the I/O response characteristic of the down-stream signaling module. We suggest that the response curve, *R*_DSP_, can be considered as a biomarker (characteristic signature) of a particular cancer signaling pathways (e.g., MAPK, PI3K/AKT, Wnt, and others (53) in specific cancer cell lines. To illustrate this, we have extracted the I/O response *R*_DSP_ of the PI3K/PTEN/AKT and MAPK pathways for different cancer cell lines using experimental data on the dose dependence of receptor activation (EGFR and PDGFR) and output signal (*p*AKT and *p*ERK) on ligand concentrations (EGF and PDGF) (49, 54, 55). The characteristic I/O response curves obtained for different activating input signals in various cell lines are shown in Figure 6. The experimental data on I/O responses were fitted by the Hill function with a Hill constant, *n*, which characterizes that response as switch-like (higher *n*) or more graded (lower *n*). As shown, AKT responses are more varied compared with the more conserved set of ERK responses that are typically switch-like (*n* = 2.65–12.8). *p*AKT responses range from switch-like for HBL and AU565 cells (Figures 6A,B) to graded hyperbolic for T47 and PE04 cells (Figures 6C,E,F) and smooth sigmoidal for MCF7 cells (Figure 6D) responses. We assume that such variety in the responses of the PI3K/PTEN/AKT pathway results from a variation in the internal parameters of the networks caused by mutations and different expression levels of signaling proteins. We suggest that I/O responses can be considered as a biomarker of mutation and protein expression status of specific cancer cells.

The *in silico* analysis of the I/O response of the PI3K/PTEN/AKT pathway confirmed that the SN can possess a smooth hyperbolic response, which corresponds to the sensitivity mode in signal transduction, and this response can be transformed into a switch-like response by changing protein parameters, which corresponds to activating mutations of the proteins involved (PTEN, PI3K, and AKT). This transformation relates to the transition from sensitivity to resistance of the SN to drug inhibiting receptor signals (32). Joint analysis of the I/O response of the PI3K/PTEN/AKT pathway and its sensitivity showed that these two key characteristics of the cellular SN are interconnected and they both significantly depend on the protein parameters and their expression levels. We observed from *in silico* experiments that perturbations of protein parameters corresponding either to 50% decreases in PTEN concentration (PTEN loss), PI3K activation mutation, or AKT overexpression significantly changes in the I/O response of the PI3K/PTEN/AKT pathway and its sensitivity. The transformation of the I/O response from smooth hyperbolic to switch-like at PTEN loss significantly endows the SN with hypersensitivity (56) that is assumed to be typical for the signaling cascade in cells at decreasing phosphatase activity (38). The steep switch response observed at PTEN loss is assumed to be due to a post-translational regulation of PTEN activity by its phosphorylation leading to the additional loss of PTEN activity (31, 48) considered in our model.

We showed that PTEN loss, or *PIK3CA* activation mutation, AKT and PI3K overexpression (40) decreased sensitivity *S*_AKT,p_ by four to six orders of magnitude for all protein parameters and this corresponds to the transformation of the I/O response from smooth hyperbolic to switch-like. At this mutation, the PI3K/PTEN/AKT pathway acquires insensitivity to further perturbation of the SN: it becomes robust in the face of both external perturbations (inhibition of input signal) and internal aberrations (mutations). PTEN loss leads to resistance to drug action (resistance to HER2 inhibition) and causes robust activation of the AKT output signal (57). Note that the observed decrease in sensitivity of the SN at either PTEN loss or PI3K aberration suggests that PTEN loss and *PIK3CA* mutation are mutually exclusive. Clinical study of oncogenic mutations in the PI3K/PTEN/AKT pathway reported both the data on mutually exclusive of PTEN loss and *PIK3CA* mutation in human breast (58) and gastric (59) carcinomas and frequently concordant in breast cancer (43, 44). As both mutations activate AKT and each decreases sensitivity of the DSP to another mutation, our finding suggests either redundancy in mutations, the need for two alterations in the single module to activate AKT, or that PTEN loss and *PIK3CA* mutation contribute differently to carcinogenesis (60).

Combining analysis of the I/O response with SA, we showed that a hyperbolic response in sensitivity mode is robust at high input (saturated receptor signals) and loses its robustness against activation mutations of the proteins at low *p*HER23 signals. Importantly, the I/O response can be modified through drug combination action, here through customization of the I/O response of the DSP by drug *D*_2_ to reach high robustness of the inhibition response to drug *D*_1_ against oncogenic protein mutations in the pathway, leading to the transformation of a hyperbolic response to a graded sigmoidal response curve (see blue lines in Figure 3A). The first benefit of that modification is an effective inhibition of output signal, *p*AKT. The most pronounced inhibition effect of the second drug is at low *p*HER23 region (0–0.2), where the sensitizing effect by the first drug, pertuzumab, is at a maximum. We exploit a synergistic interaction between these two drugs, specifically the sensitizing effect of one drug on the action of another. A second benefit of that modification is a decrease in the sensitivity of this response in the range of low *p*HER23 signal (0–0.2) (see blue lines in Figure 3A). We hypothesized that the modified response is more robust against oncogenic mutation than the hyperbolic type of the response. To check this, we carried out robustness screening of this response with respect to the following aberrations in the PI3K/PTEN/AKT pathway: PTEN loss, activation mutation of PI3K, and overexpression of AKT. In all these cases we observed effective inhibition of *p*AKT and so a high degree of robustness in the modified response to common oncogenic mutations in the PI3K/PTEN/AKT pathway (see green lines in Figures 3A,C). We showed that a hyperbolic response is more adapted to signal discrimination and signal transaction while a modified graded sigmoidal response close to linear response is more adapted to therapeutic inhibition of signaling pathway activated by oncogenic mutations.

Note, a similar transformation from hyperbole to switch-like response was observed *in silico* and *in vitro* experiments in Raf/MEK/ERK pathway as a result of deletion of negative feedbacks from ppERK to Son of Sevenless protein (SOS) and Raf (26). This finding and other theoretical and experimental studies show that multiple feedback and gene regulation can significantly control drug efficacy (4, 5, 52). An extension of the modularity approach used in our work should be performed to explore the consequences of feedback and gene regulation networks, which control the robustness of SNs in normal and malignant conditions (61, 62). For example, to develop more reliable model of SN response to drug combination, it is necessary to take into account the negative feedback in the PI3K/PTEN/AKT pathway which includes phosphorylation (inactivation) of GSK3β by *p*AKT which phosphorylates (inactivates) PTEN, and therefore increases AKT phosphorylation (31).

As discussed in the Section “Introduction,” many experiments and clinical trials confirm the suppression of drug resistance and the robustness response of drug combinations acting on the vertical signaling pathways in different cancers. For example, complementary inhibition of the vertical targets in the PI3K/PTEN/AKT/mTOR pathway restores the inhibition effect of trastuzumab and pertuzumab (15, 17). Moreover, the experimental study of the combination of PI3K inhibition (GDC-0941) with HER2 inhibitors (trastuzumab, pertuzumab) showed that the high efficacy of this combination therapy is a general effect for different breast cancer cell lines despite the fact that different cell lines have different susceptibilities/resistance to each drug separately (42).

Our *in silico* experiments indicate that anti-cancer response robustness to drug action emerges due to a smooth sigmoid response of the SN when modified by drug combination. Experimentally, the robustness of the modified response to a change in expression level of signaling proteins can be measured in RNA interference (RNAi) screening with respect to repression of protein expression. RNAi screening has been applied successfully to a study of resistance mechanisms and development of effective combination therapy by determining drug targets (60, 63), and allows estimation of the robustness of the therapeutic response to the perturbations in expression of the proteins (isomers or catalytic/regulatory protein subunits) surrounding drug targets in a SN (both vertical and horizontal targeted pathways). Robustness screening with respect to overexpression of the proteins surrounding targeted proteins can be measured using isogenic cell clones customized to genomic status involved in screening (64). Most critical experimental validations are assumed to be carried out with heterogeneous tumor samples. Here we suggest that designing into a combination therapy regime, an unchanged landscape of sensitivity across a mutation spectrum, such as that shown in heatmap in Figure 4 (columns 5, 6, and 8), may slow down the evolution of subpopulations of cancer cells under a selection pressure of drug therapy and suppresses the development of drug-resistant clones (28, 65). Further, effecting this slowing down in resistance evolution may require a longer-term engineered drug combination regime that accounts for a drift in cell signaling behavior over many cellular generations.

The method of investigation of the I/O response of the SN modules developed here can be considered as a supplementary tool for the analysis of the dose dependence for drug *D*_1_ and the mechanism of IC_50_ changes at oncogenic mutations and combination therapy. To show the link between response dynamics properties of the PI3K/PTEN/AKT pathway and the dose response of the whole SN, we calculated the dose dependence of *p*AKT on concentration of pertuzumab (drug *D*_1_): *R*_SN_ = *C*_out_ (*C*_in_, *D, P*_SN_, *t*), and showed how the modification of the DSP response by mutations and drugs changes IC_50_ for pertuzumab (Figure 5). In the calculation, we observed a decrease of IC_50_ for pertuzumab in the presence of PI3K inhibitor, LY294002, suggesting lower concentrations to be effective. These results supplement the analysis of the drug-induced shift in dose dependence and the synergetic effect of two drugs targeting vertical pathways discussed by Fitzgerald et al. (52). Additionally, we showed that drug-induced sensitivity to the second drug modify both response curve and dose dependence for the first drug that significantly enhances synergetic effect of two drugs in combination.

We showed that the sensitivity of the DSP without customization increases significantly in the range of IC_80_ of pertuzumab (see solid and dashed black lines in Figure 5). The simplest way to reach the goal of high inhibition and low sensitivity is to increase the drug concentration and go far beyond the range of maximal sensitivity (>1 μM pertuzumab, Figure 5). In practice, this is often not a realistic solution for toxicity reasons, although there is evidence that drug concentrations that are twice the IC_50_ can suppress acquired resistance due to mutations following drug therapy (29). A more realistic approach is to customize the network response to inhibition through combination therapy, manipulating the inhibition and sensitivity dose dependencies through the action of the second drug, which modifies the I/O response of the DSP. Additionally, our calculation showed that a significantly lower IC_50_ for pertuzumab in combination with the PI3K inhibitor can be a basis for decreasing a dose of drug *D*_1_ to reduce its toxicity.

We applied the method to two well-known targets and drugs and investigated the mechanism of their effectiveness. We demonstrated that the drug-induced sensitivity of the DSP to changes in protein parameters and their expression levels has the potential to sensitize this pathway to mutations that compensate for drug action, i.e., low output signal at inhibited input signal. Moreover, such mutations can effect a decrease in sensitivity of the DSP to further external and internal perturbations leading to an increase in DSP robustness. It is thus possible that drug action may significantly perturb DSP functioning, stimulating the activation of compensatory response mechanisms in the cell.

We then demonstrated a method of minimizing the drug-sensitizing effect while maintaining the intended effect of drug action by customizing the I/O response of the DSP response. The goal of this customization was to reach a high inhibition of input signal without sensitizing the SN to mutations leading to an increased potential for resistance, specifically PTEN loss and AKT overexpression. Our focus was the modification of the I/O response of the PI3K/PTEN/AKT pathway to inhibition of pHER2 by pertuzumab.

These results suggest that the I/O response of signaling modules can be used as biomarker to select advantaged drug targets for mono- and combination therapy. To illustrate this concept, we considered different strategies of drug selection depending on the response of the PI3K/PTEN/AKT signaling module of the different cells represented in Figure 6. We assume that in the case of switch-like response with low threshold of activation (low parameter *K*) such as represented in Figure 6A (HBL cells) and Figure 6B (AU565 cells), a rational strategy is to use drug combination in which one drug targets the DSP and modifies the response curve to more graded or sigmoid form while another drug in combination inhibits input and output signals according to that modified response curve. In the case of responses of smooth hyperbolic type like represented in Figure 6C (T47), Figure 6E (PE04), and Figure 6F (NIH 3T3), the drug targeting USP effectively inhibits *p*AKT in the 80–90% range of inhibited receptor signal but sensitizes down-stream module to compensation mutations. To avoid the latter effect, we suggest modifying the response of the down-stream module to a smooth sigmoid curve by a second drug targeting the DSP. The I/O smooth sigmoid response of the type given in Figure 6D (MCF7) is assumed to endow the signaling module with high sensitivity to the drug inhibiting its input signal and low sensitivity to compensatory mutations. In this case, inhibition of the USP by one drug is effective with respect to both signal inhibition and sensitivity suppression. The use of the second drug targeting down-stream pathway is redundant from the viewpoint of the response modification and toxicity. These analyses indicate the strategy of drug–diagnostic co-development (2, 66) when drug therapies (mono- or combination) are selected based on the integrative biomarker corresponding to the I/O response of signaling modules. Stratification of patients according to different I/O response curves can help identify patients who are most likely to benefit from selected therapy. Constant monitoring of the transformation of the I/O response curve during treatment can indicate at compensatory gene regulation (12, 45, 67, 68) and acquired mutations as a result of the selected therapy and so can serve as a guide for changing therapeutic regime. The obtained results showed that it is desirable to introduce an additional characteristic of drugs to support this strategy – drug-induced sensitivity of the SN or resistance potential of drugs, which significantly depends on the I/O response of targeted SN.

The joint analysis, the I/O response of the PI3K/PTEN/AKT pathway, and *p*AKT dose response for pertuzumab revealed the challenge of the trade-off between high sensitivity to drug action and oncogenic mutations. The proposed method of modification of the I/O response of the PI3K/PTEN/AKT pathway and manipulating separately by the inhibition and sensitivity dose dependencies through action of a second drug resolved this problem partially. Despite PI3K inhibition causing the loss of relative sensitivity at different perturbations (pHER2 inhibition and the different mutations in PI3K/PTEN/AKT pathway), it increased the overall level of sensitivity of the DSP to protein parameters that is assumed to sensitize the SN to the compensation mutations (compare columns 1 and 5 in Figure 4). To further optimize this trade-off at the combination design stage, we propose that other means of customizing the I/O response to improve drug resistance potential are needed, e.g., by using the methods and results of synthetic biology in signaling pathway engineering to customize their I/O responses (69–71). For example, using some modulators it is possible to alter/reshape dose dependence from a graded to a sharply sensitive, switch-like response and a time dependence from sustained to pulse or delayed responses in MAPK pathway signaling (71). It has been shown that modification of cellular response obtained by genetic engineering can also be reached through drug action. For example, cellular responses were engineered by dynamic rewiring of SN topology (22, 72) and controlling negative feedback circuits in SN by drug combination (26). Ultimately the application of this arsenal of engineering methods in network and synthetic biology, underpinned by integrative systems biology, can be a powerful tool for adaptation of signaling response to effective combination therapy and rational drug combinations co-development.

## Conflict of Interest Statement

The authors declare that the research was conducted in the absence of any commercial or financial relationships that could be construed as a potential conflict of interest.

## Supplementary Material

The Supplementary Material for this article can be found online at http://www.frontiersin.org/Journal/10.3389/fonc.2014.00013/abstract
