# Supplementary material for: Customizing the Therapeutic Response of Signaling Networks to Promote Antitumor Responses by Drug Combinations
Source: Front Oncol. 2014 Feb 5;4:13. doi: 10.3389/fonc.2014.00013 (PMC3914444; doi:10.3389/fonc.2014.00013)
Supplement: Supplementary file 1 [file 71980_Goltsov_DataSheet1.PDF]

Supplementary Information

1. Mathematical model of Ras/RAF/MEK/ERK and PI3K/PTEN/AKT pathway

System of ordinary differential equations

$$\frac{d[\mathrm{E3}]}{dt} = \text{-}\mathrm{V}_1 \quad \text{(S1.1)}$$

$$\frac{d[\mathrm{E3H}]}{dt} = \mathrm{V}_1\text{-}\mathrm{V}_2 - \mathrm{V}_{51} \quad \text{(S1.2)}$$

$$\frac{d[\mathrm{HRG}]}{dt} = \text{-}\mathrm{V}_1 \quad \text{(S1.3)}$$

$$\frac{\mathrm{d}[\mathrm{E3H_c}]}{\mathrm{dt}} = \mathrm{V}_{51}\text{-}\mathrm{V}_{52} \quad \text{(S1.4)}$$

$$\frac{d[\mathrm{E2}]}{dt} = \text{-}\mathrm{V}_2\text{-}\mathrm{V}_{49}\text{-}\mathrm{V}_{52} \quad \text{(S1.5)}$$

$$\frac{d[\mathrm{E23H}]}{dt} = \mathrm{V}_2\text{-}\mathrm{V}_3+\mathrm{V}_4+\mathrm{V}_{52}\text{-}\mathrm{V}_{53} \quad \text{(S1.6)}$$

$$\frac{d[\mathrm{E23H_c}]}{dt} = \mathrm{V}_{53}\text{-}\mathrm{V}_{54} \quad \text{(S1.7)}$$

$$\frac{d[\mathrm{pE23H}]}{dt} = \mathrm{V}_3\text{-}\mathrm{V}_4\text{-}\mathrm{V}_5+\mathrm{V}_8\text{-}\mathrm{V}_{27}+\mathrm{V}_{29}\text{-}\mathrm{V}_{48}+\mathrm{V}_{54} \quad \text{(S1.8)}$$

$$\frac{d[\mathrm{Shc}]}{dt} = \text{-}\mathrm{V}_5+\mathrm{V}_{10} \quad \text{(S1.9)}$$

$$\frac{d[\mathrm{pE23H\text{-}Shc}]}{dt} = \mathrm{V}_5\text{-}\mathrm{V}_6 \quad \text{(S1.10)}$$

$$\frac{d[\mathrm{pE23H\text{-}pShc}]}{dt} = \mathrm{V}_6\text{-}\mathrm{V}_7 \quad \text{(S1.11)}$$

$$\frac{d[\mathrm{GS}]}{dt} = \text{-}\mathrm{V}_7+\mathrm{V}_9 \quad \text{(S1.12)}$$

$$\frac{d[\mathrm{Shc\text{-}GS}]}{dt} = \mathrm{V}_8\text{-}\mathrm{V}_9 \quad \text{(S1.13)}$$

$$\frac{d[\mathrm{pShc}]}{dt} = \mathrm{V}_9\text{-}\mathrm{V}_{10} \quad \text{(S1.14)}$$

$$\frac{d[\mathrm{pE23H\text{-}pShc\text{-}GS}]}{dt} = \mathrm{V}_7\text{-}\mathrm{V}_8 \quad \text{(S1.15)}$$

$$\frac{d[\mathrm{ppAkt\text{-}PIP3}]}{dt} = \mathrm{V}_{44}\text{-}\mathrm{V}_{45} \quad \text{(S1.16)}$$

$$\frac{d[\mathrm{Ras\text{-}GDP}]}{dt} = \text{-}\mathrm{V}_{11}+\mathrm{V}_{12} \quad \text{(S1.17)}$$

$$\frac{d[\mathrm{Ras\text{-}GTP}]}{dt} = \mathrm{V}_{11}\text{-}\mathrm{V}_{12} \quad \text{(S1.18)}$$

$$\frac{d[\mathrm{Raf}]}{dt} = \text{-}\mathrm{V}_{13}+\mathrm{V}_{14} \quad \text{(S1.19)}$$

$$\frac{d[\text{Raf*}]}{dt} = \text{V}_{13}\text{-V}_{14} \tag{S1.20}$$

$$\frac{d[\text{MEK}]}{dt} = \text{-V}_{15}+\text{V}_{18} \tag{S1.21}$$

$$\frac{d[\text{pMEK}]}{dt} = \text{V}_{15}\text{-V}_{16}\text{-V}_{19}+\text{V}_{22} \tag{S1.22}$$

$$\frac{d[\text{PP2A}]}{dt} = \text{-V}_{16}+\text{V}_{18}\text{-V}_{20}+\text{V}_{22}\text{-V}_{41}+\text{V}_{43}\text{-V}_{45}+\text{V}_{47} \tag{S1.23}$$

$$\frac{d[\text{MEKP-PP2A}]}{dt} = \text{V}_{16}\text{-V}_{17}+\text{V}_{21}\text{-V}_{22} \tag{S1.24}$$

$$\frac{d[\text{MEK-PP2A}]}{dt} = \text{V}_{17}\text{-V}_{18} \tag{S1.25}$$

$$\frac{d[\text{pAkt-PIP3-PP2A}]}{dt} = \text{V}_{41}\text{-V}_{42}+\text{V}_{46}\text{-V}_{47} \tag{S1.26}$$

$$\frac{d[\text{ppMEKPP-PP2A}]}{dt} = \text{V}_{20}\text{-V}_{21} \tag{S1.27}$$

$$\frac{d[\text{ppMEKPP-PP2A}]}{dt} = \text{V}_{20}\text{-V}_{21} \tag{S1.28}$$

$$\frac{d[\text{ppMEK}]}{dt} = \text{V}_{19}\text{-V}_{20} \tag{S1.29}$$

$$\frac{d[\text{ERK}]}{dt} = \text{-V}_{23}+\text{V}_{24} \tag{S1.30}$$

$$\frac{d[\text{pERKP}]}{dt} = \text{V}_{23}\text{-V}_{24}\text{-V}_{25}+\text{V}_{26} \tag{S1.31}$$

$$\frac{d[\text{ppERK}]}{dt} = \text{V}_{25}\text{-V}_{26} \tag{S1.32}$$

$$\frac{d[\text{ppAkt-PIP3-PP2A}]}{dt} = \text{V}_{45}\text{-V}_{46} \tag{S1.33}$$

$$\frac{d[\text{Akt-PIP3}]}{dt} = \text{V}_{39}\text{-V}_{40}+\text{V}_{43} \tag{S1.34}$$

$$\frac{d[\text{PI3K}]}{dt} = -\text{V}_{27}+\text{V}_{30}-\text{V}_{58} \tag{S1.35}$$

$$\frac{d[\text{pE23H-PI3K}]}{dt} = \text{V}_{27}\text{-V}_{28} \tag{S1.36}$$

$$\frac{d[\text{pE23H-PI3K*}]}{dt} = \text{V}_{28}\text{-V}_{29} \tag{S1.37}$$

$$\frac{d[\text{PI3K*}]}{dt} = \text{V}_{29}\text{-V}_{30}\text{-V}_{31}+\text{V}_{56} \tag{S1.38}$$

$$\frac{d[\text{PI}]}{dt} = \text{-V}_{31}+\text{V}_{34} \tag{S1.39}$$

$$\frac{d[\text{PTEN}]}{dt} = -\text{V}_{32}+\text{V}_{34}-\text{V}_{35}-\text{V}_{36}+2\text{V}_{38}-\text{V}_{57} \tag{S1.40}$$

$$\frac{d[\text{PIP3}]}{dt} = \text{-V}_{32}\text{-V}_{39}+\text{V}_{56} \tag{S1.41}$$

$$\frac{d[\text{PTEN-PIP3}]}{dt} = \text{V}_{32}\text{-V}_{33} \quad \text{(S1.42)}$$

$$\frac{d[\text{PTEN-PI}]}{dt} = \text{V}_{33}\text{-V}_{34} \quad \text{(S1.43)}$$

$$\frac{d[\text{pPTEN}]}{dt} = \text{V}_{35}\text{-V}_{36} \quad \text{(S1.44)}$$

$$\frac{d[\text{pPTENP-PTEN}]}{dt} = \text{V}_{36}\text{-V}_{37} \quad \text{(S1.45)}$$

$$\frac{d[\text{PTEN-PTEN}]}{dt} = \text{V}_{37}\text{-V}_{38} \quad \text{(S1.46)}$$

$$\frac{d[\text{PI3K*-PI}]}{dt} = \text{V}_{31}\text{-V}_{55} \quad \text{(S1.47)}$$

$$\frac{d[\text{PI3K*-PIP3}]}{dt} = \text{V}_{55}\text{-V}_{56} \quad \text{(S1.48)}$$

$$\frac{d[\text{Akt}]}{dt} = \text{-V}_{39} \quad \text{(S1.49)}$$

$$\frac{d[\text{pAkt-PIP3}]}{dt} = \text{V}_{40}\text{-V}_{41}\text{-V}_{44}+\text{V}_{47} \quad \text{(S1.50)}$$

$$\frac{d[\text{Akt-PIP3-PP2A}]}{dt} = \text{V}_{42}\text{-V}_{43} \quad \text{(S1.51)}$$

$$\frac{d[2C4]}{dt} = \text{-V}_{49} \quad \text{(S1.52)}$$

$$\frac{d[\text{E2-2C4}]}{dt} = \text{V}_{49}\text{-V}_{50} \quad \text{(S1.53)}$$

$$\frac{d[\text{E2-2C4}_{\text{c}}]}{dt} = \text{V}_{50} \quad \text{(S1.54)}$$

$$\frac{d[\text{PTEN-bpV}]}{dt} = \text{V}_{57} \quad \text{(S1.55)}$$

$$\frac{d[\text{PI3K-LY}]}{dt} = \text{V}_{58} \quad \text{(S1.56)}$$

where reaction rates V_i are determined by the following equations:

$$\text{V}_1=\text{k}_1\cdot([\text{E3}]\cdot[\text{HRG}]-\text{K}_{\text{d},1}\cdot[\text{E3H}]) \quad \text{(S1.57)}$$

$$\text{V}_2=\text{k}_2\cdot([\text{E3H}]\cdot[\text{E2}]-\text{K}_{\text{d},2}\cdot[\text{E23H}]) \quad \text{(S1.58)}$$

$$\text{V}_3=\text{k}_3\cdot[\text{E23H}]/(\text{K}_{\text{d},3}+[\text{E23HP}]) \quad \text{(S1.59)}$$

$$\text{V}_4=\text{V}_{\text{max},4}\cdot[\text{pE23H}]\,/\,(\text{K}_{\text{m},4}+[\text{pE23H}]) \quad \text{(S1.60)}$$

$$\text{V}_5=\text{k}_5\cdot([\text{pE23H}]\cdot[\text{Shc}]-\text{K}_{\text{d},5}\cdot[\text{pE23H-Shc}]) \quad \text{(S1.61)}$$

$$\text{V}_6=\text{k}_6\cdot[\text{pE23H-Shc}]-\text{k}_{-6}\cdot[\text{pE23H-pShc}] \quad \text{(S1.62)}$$

$$V_7 = k_7 \cdot ([\text{pE23H-pShc}] \cdot [\text{GS}] - K_{d,7} \cdot [\text{pE23H-pShc-GS}]) \quad \text{(S1.63)}$$

$$V_8 = k_8 \cdot ([\text{pE23H-Shc-GS}] - K_{d,8} \cdot [\text{pE23HP}] \cdot [\text{Shc-GS}]) \quad \text{(S1.64)}$$

$$V_9 = k_9 \cdot [\text{Shc-GS}] - k_{-9} \cdot [\text{pShc}] \cdot [\text{GS}] \quad \text{(S1.65)}$$

$$V_{10} = V_{max,10} \cdot [\text{pShc}] \,/\, (K_{m,10} + [\text{pShc}]) \quad \text{(S1.66)}$$

$$V_{11} = k_{11} \cdot [\text{Ras-GDP}] \cdot [\text{Shc-GS}]/(K_{m,11} + [\text{Ras-GDP}]) \quad \text{(S1.67)}$$

$$V_{12} = V_{12} \cdot [\text{Ras-GTP}]/(K_{m,12} + [\text{Ras-GTP}]) \quad \text{(S1.68)}$$

$$V_{13} = k_{13} \cdot [\text{Raf}] \cdot [\text{Ras-GTP}]/(K_{m,13} + [\text{Raf}]) \quad \text{(S1.69)}$$

$$V_{14} = k_{14} \cdot [\text{Raf*}] \cdot ([\text{ppAkt-PIP3}] + [E_{Raf}])/([\text{Raf*}] + K_{m,14}) \quad \text{(S1.70)}$$

$$V_{15} = k_{15} \cdot [\text{MEK}] \cdot [\text{Raf*}]/(K_{m,15} + [\text{MEK}]) \quad \text{(S1.71)}$$

$$V_{16} = k_{16} \cdot [\text{pMEK}] \cdot [\text{PP2A}] \quad \text{(S1.72)}$$

$$V_{17} = k_{cat,16} \cdot [\text{pMEK-PP2A}] \quad \text{(S1.73)}$$

$$V_{18} = k_{18} \cdot [\text{MEK-PP2A}] \quad \text{(S1.74)}$$

$$V_{19} = k_{15} \cdot [\text{pMEK}] \cdot [\text{Raf*}]/(K_{m,15} + [\text{pMEK}]) \quad \text{(S1.75)}$$

$$V_{20} = k_{16} \cdot ([\text{PP2A}] \cdot [\text{ppMEK}] - K_{d,16} \cdot [\text{ppMEK-PP2A}]) \quad \text{(S1.76)}$$

$$V_{21} = k_{cat,16} \cdot [\text{ppMEK-PP2A}] \quad \text{(S1.77)}$$

$$V_{22} = k_{22} \cdot [\text{pMEK-PP2A}] \quad \text{(S1.78)}$$

$$V_{23} = k_{23} \cdot [\text{ERK}] \cdot [\text{ppMEK}]/(K_{m,23} + [\text{ERK}]) \quad \text{(S1.79)}$$

$$V_{24} = V_{max,24} \cdot [\text{pERK}]/(K_{m,24} + [\text{pERK}]) \quad \text{(S1.80)}$$

$$V_{25} = k_{23} \cdot [\text{ppMEK}] \cdot [\text{pERK}]/(K_{m,23} + [\text{pERK}]) \quad \text{(S1.81)}$$

$$V_{26} = V_{max,24} \cdot [\text{ppERK}]/(K_{m,24} + [\text{ppERK}]) \quad \text{(S1.82)}$$

$$V_{27} = k_{27} \cdot ([\text{pE23H}] \cdot [\text{PI3K}] - K_{d,27} \cdot [\text{pE23H-PI3K}]) \quad \text{(S1.83)}$$

$$V_{28}=k_{28}\cdot[\text{pE23H-PI3K}] \tag{S1.84}$$

$$V_{29}=k_{29}\cdot[\text{pE23H-PI3K*}] \tag{S1.85}$$

$$V_{30}=k_{30}\cdot[\text{pE23H-PI3K}] \tag{S1.86}$$

$$V_{31}=k_{31}\cdot([\text{PI}]\cdot[\text{pE23H-PI3K}]-K_{d,31}\cdot[\text{PI3K*-PI}]) \tag{S1.87}$$

$$V_{32}=k_{32}\cdot([\text{PIP3}]\cdot[\text{PTEN}]-K_{d,32}\cdot[\text{PTEN-PIP3}]) \tag{S1.88}$$

$$V_{33}=k_{33}\cdot[\text{PTEN-PIP3}] \tag{S1.89}$$

$$V_{34}=k_{34}\cdot[\text{PTEN-PI}] \tag{S1.90}$$

$$V_{35}=V_{max,35}\cdot[\text{PTEN}]/(K_{m,35}+[\text{PTEN}]) \tag{S1.91}$$

$$V_{36}=k_{36}\cdot([\text{PTEN}]\cdot[\text{pPTEN}]-K_{d,36}\cdot[\text{pTENP-PTEN}]) \tag{S1.92}$$

$$V_{37}=k_{cat,37}\cdot[\text{pPTENP-PTEN}] \tag{S1.93}$$

$$V_{38}=k_{38}\cdot[\text{PTEN-PTEN}] \tag{S1.94}$$

$$V_{39}=k_{39}\cdot([\text{PIP3}]\cdot[\text{Akt}]-K_{d,39}\cdot[\text{Akt-PIP3}]) \tag{S1.95}$$

$$V_{40}=V_{max,40}\cdot[\text{Akt-PIP3}]/(K_{m,40}+[\text{Akt-PIP3}]) \tag{S1.96}$$

$$V_{41}=k_{41}\cdot([\text{pAkt-PIP3}]\cdot[\text{PP2A}]-K_{d,41}\cdot[\text{ppAkt-PIP3-PP2A}]) \tag{S1.97}$$

$$V_{42}=k_{cat,42}\cdot[\text{pAkt-PIP3-PP2A}] \tag{S1.98}$$

$$V_{43}=k_{43}\cdot[\text{Akt-PIP3-PP2A}] \tag{S1.99}$$

$$V_{44}=V_{max,40}\cdot[\text{pAkt-PIP3}]/(K_{m,40}+[\text{pAkt-PIP3}]) \tag{S1.100}$$

$$V_{45}=k_{45}\cdot([\text{ppAkt-PIP3}]\cdot[\text{PP2A}]-K_{d,45}\cdot[\text{ppAkt-PIP3-PP2A}]) \tag{S1.101}$$

$$V_{46}=k_{cat,46}\cdot[\text{ppAkt-PIP3-PP2A}] \tag{S1.102}$$

$$V_{47}=k_{47}\cdot[\text{pAkt-PIP3-PP2A}] \tag{S1.103}$$

$$V_{48}=k_{48}\cdot[\text{pE23H}] \tag{S1.104}$$

$$V_{49}=k_{49}\cdot([2C4]\cdot[E2]-K_{d,49}\cdot[E2\text{-}2C4]) \quad (S1.105)$$

$$V_{50}=k_{50}\cdot[E2\text{-}2C4]-k_{-50}\cdot[E2\text{-}2C4_c] \quad (S1.106)$$

$$V_{51}=k_{51}\cdot[E3H] \quad (S1.107)$$

$$V_{52}=k_{2}\cdot([E3H_c]\cdot[E2]-K_{d,2}\cdot[E23H]) \quad (S1.108)$$

$$V_{53}=k_{53}\cdot[E23H] \quad (S1.109)$$

$$V_{54}=k_{3}\cdot([E23H_c]-K_{d,3}\cdot[pE23H]) \quad (S1.110)$$

$$V_{55}=k_{55}\cdot[PI3K^{*}\text{-}PI] \quad (S1.111)$$

$$V_{56}=k_{56}\cdot[PI3K^{*}\text{-}PIP3] \quad (S1.112)$$

$$V_{57}=k_{57}\cdot([PTEN]\cdot[bpV]-K_{d,57}\cdot[PTEN\_bpV]) \quad (S1.113)$$

$$V_{58}=k_{58}\cdot([PI3K]\cdot[LY]-K_{d,58}\cdot[PI3K\_LY]) \quad (S1.114)$$

Table S1. Abbreviations used in the model

Abbreviations in text, ODEs, schemes	Protein names	Abbreviations in SBML file
E2, HER2	ErbB2 receptor	E2
E3, HER3	ErbB3 receptor	E3
HRG	Heregulin	HRG
E3H	ErbB3/HRG ligand/receptor complex	E3H
$E3H_c$	ErbB3/HRG ligand/receptor complex	E3H_C
E23H	Heterodimer of ErbB3/HRG with ErbB2	E23H
$E23H_c$	Heterodimer of ErbB3/HRG with ErbB2	E23H_C
pE23H, pHER2	Phosphorylated heterodimer of ErbB3/HRG with ErbB2	E23HP
Grb2	growth factor receptor-binding protein 2	
Ras-GDP	Ras-GDP protein	RasGDP
Ras-GTP	Ras-GTP protein	RasGTP
Raf	Raf protein	Raf
Raf*	Activated Raf	Rafa
PTEN	Phosphatase and tensinhomologue deleted on chromosome ten	PTEN
pPTEN	Phosphorylated PTEN	PTENP
AKT	AKT protein	Akt
2C4	Pertuzumab	Per
MAPK	Mitogen-activated protein kinase	
MEK	MAPK/ERK kinase	
pMEK	Phosphorylated MEK	MEKP
ppMEK	Doubly phosphorylated MEK	MEKPP
ERK	extracellular signal-regulated kinase	
pERK	Phosphorylated ERK	ERKP
ppERK	Doubly phosphorylated ERK	ERKPP
MKP3	MAPK phosphatase 3	
PDK1	3-phosphoinositide-dependent kinase 1	
PI	phosphatidylinositol	PI
PIP3	phosphatidylinositol-3,4,5-trisphosphate	
PI3K	phosphatidylinositol 3’-kinase	PI3K
PI3K*	Activated PI3K	PI3Ka
PP2A	protein phosphatase 2A	PP2A
Shc	Srchomology and collagen domain protein	Shc
pShc	Phosphorylated Shc	ShcP
Sos	Son of Sevenless homologue protein	
GS	Grb2–Sos complex	GS
pE23H-Shc	Complex of pE23H with Shc	E23HP_Shc
pE23H-pShc	Complex of pE23H with pShc	E23HP_ShP
Shc-GS	Complex of Shc with GS	ShGS
pE23H-pShc-GS	Complex of pE23H-pShc with GS	E23HP_ShGS
AKT-PIP3	Complex of AKT with PIP3	AKT_PIP3
pAKT-PIP3	Complex of pAKT with PIP3	AKT_PI_P

ppAKT-PIP3	Complex of ppAKT with PIP3	AKT_PI_PP
ppAKT-PIP3-PP2A	Complex of ppAKT-PIP3 with PP2A	AKT_PI_PP_PP2A
AKT-PIP3-PP2A	Complex of AKT-PIP3 with PP2A	AKT_PIP3_PP2A
pAKT-PIP3-PP2A	Complex of pAKT-PIP3 with PP2A	AKT_PI_P_PP2A
E2-2C4	Complex of E2 with 2C4	E2_Per
E2-$2C4_c$	Complex of E2 with 2C4	E2Per
PTEN-PTEN	Complex of PTEN with PTEN	PTEN_PTEN
pPTEN-PTEN	Complex of pPTEN withPTEN	PTENP_PTEN
PTEN-PIP3	Complex of PTEN with PIP3	PTEN_PIP3
PTEN-PI	Complex of PTEN with PI	PTEN_PI
pMEKP-PP2A	Complex of pMEKP with PP2A	MEKP_PP2A
MEK-PP2A	Complex of MEK with PP2A	MEK_PP2A
ppMEKPP-PP2A	Complex of ppMEKPP with PP2A	MEKPP_PP2A
pE23H-PI3K	Complex of pE23H with PI3K	E23HP_PI3K
PI3K*-PI	Complex of PI3K* with PI	PI3Ka_PI
pE23H-PI3K*	Complex of pE23H with PI3K*	E23HP_PI3Ka
E_{Raf}	Phosphatase dephosphorylating Raf*	E_Raf
bpV	Bisperoxovanadium compound, bpV(pic), PTEN inhibitor	bpV
LY	LY294002, PI3K inhibitor	LY
PTEN-bpV	Enzyme-inhibitor complex of PTEN and bpV(pic)	PTEN_bpV
PI3K-LY	Enzyme-inhibitor complex of PI3K and LY294002	PI3K_LY

Table S2. Kinetic parameters of the model. k_i , k_{-i} – rate constants of forward and reverse reactions ($nM^{-1}\ min^{-1}$, min^{-1}); $K_{d,i}$, $K_{m,i}$ – dissociation and Michaelis constants (nM).

Reaction rate	Kinetic parameters	Values in our model	Remarks	Values from (1)	Values from (2)
**HER3 and HER2 binding**					
V_1	k_1	0.005	estimation	0.001	0.003
	$K_{d,1}$	600; 0.2[1)]	estimation	0.6	20
V_{51}	k_{51}	0.01	estimation		
V_2	k_2	10	estimation	0.01	0.01
V_{52}	$K_{d,2}$	10	estimation	10	10
V_{53}	k_{53}	0.01	estimation		
V_3	k_3	1	estimation	1	1
	$K_{d,3}$	0.1	estimation	0.01	0.01
V_4	$V_{max,4}$	10	estimation	62	450
	$K_{m,4}$	50	estimation	50	50
**Shc and GS binding**					
V_5	k_5	0.06	estimation	0.1	0.09
	$K_{d,5}$	1	estimation	1	6
V_6	k_6	12	estimation	20	6
	k_{-6}	3	estimation	5	0.06
V_7	k_7	36	estimation	60	0.009
	$K_{d,7}$	9	estimation	9	4.3
V_8	k_8	12	estimation	2040	0.12
	$K_{d,8}$	0.1	estimation	7.8	0.002
V_9	k_9	35	estimation	40.8	0.1
	k_{-9}	0	estimation	0	0.2
V_{10}	$V_{max,10}$	0.0154	estimation	0.0154	1.7
	$K_{m,10}$	340	estimation	340	340
**E23HP binding with PI3K and PI3K activation**					
V_{27}	k_{27}	3	estimation	0.1	
	$K_{d,27}$	1	estimation	20	
V_{28}	k_{28}	300	estimation	9.85	
	k_{-28}	0	estimation	0.1	
V_{29}	k_{29}	13500	estimation	45.8	
	k_{-29}	0	estimation	0.047	
V_{30}	V_{30}	900	estimation	$V_{m,26}$=2620	
**Ras/Raf/MEK/ERK cascade**					
V_{11}	k_{11}	6	estimation	0.22	
	$K_{m,11}$	0.18	estimation	0.18	
V_{12}	$V_{max,12}$	3	estimation	0.3	
	$K_{m,12}$	0.1	estimation	0.06	
V_{13}	k_{13}	1	estimation	1.53	
	$K_{m,13}$	11.7	estimation	11.7	
V_{14}	k_{14}	0.6	estimation	$6.7\ 10^{-3}$	
	$K_{m,14}$	30	estimation	8	
V_{15} V_{19}	k_{15}	2.1	estimation	3.5	

	$K_{m,15}$	1	estimation	317	
V_{16}	k_{16}	0.06	estimation		
V_{17} ,V_{21}	$k_{cat,16}$	0.6	estimation		
V_{18}	k_{18}	3	estimation		
V_{20}	k_{16}	0.06	estimation		
	$K_{d,16}$	1	estimation		
V_{22}	k_{22}	0.06	estimation		
V_{23} V_{25}	k_{23}	1.2	estimation	9.5	
	$K_{m,23}$	10	estimation	$1.4\ 10^5$	
V_{24} V_{26}	$V_{max,24}$	1.8	estimation	0.3	
	$K_{m,24}$	10	estimation	160	
**PIP3 $\rightarrow$ PI**					
V_{31}	k_{31}	0.03	estimation		
	$K_{d,31}$	140	estimation	K_m =40	
V_{55}	k_{55}	30	estimation	k_{27} =16.9	
V_{56}	k_{56}	30	estimation		
V_{32}	k_{32}	8000	estimation		
	$K_{d,32}$	0.01	estimation	$K_{m,28}$=9	
V_{33}	k_{33}	15±5	estimation		
V_{34}	k_{34}	3.6	estimation		
**PTEN $\rightarrow$ pPTEN**					
V_{35}	$V_{max,35}$	150	estimation		
	$K_{m,35}$	2	estimation		
V_{36}	k_{36}	1	estimation		
	$K_{d,36}$	2.2	estimation		
V_{37}	k_{37}	150	estimation		
V_{38}	k_{38}	150	estimation		
**AKT activation pathway**					
V_{39}	k_{39}	15000	estimation	507	
	$K_{d,39}$	20	estimation	234	
V_{40} V_{44}	$V_{max,40}$	$1.5 \cdot 10^4$	estimation	$2 \cdot 10^4$	
	$K_{m,40}$	0.1	estimation	$8 \cdot 10^4$	
V_{41}	k_{41}	3	estimation		
V_{42}, V_{46}	k_{42}	45	estimation		
V_{43}	k_{43}	30	estimation		
V_{45}	k_{41}	3	estimation		
	$K_{d,41}$	0.1	estimation		
V_{47}	k_{47}	0.3	estimation		
**Receptor internalization**					
V_{48}	k_{48}	0.001	estimation	0.001	
**2C4 binding**					
V_{49}	k_{49}	0.003	estimation		
	$K_{d,49}$	$2 \cdot 10^4$; 7[1)]	estimation, K_d=8.5 nm (3)		
V_{50}	k_{50}	0.6	estimation		
	k_{-50}	0.012	estimation		

PTEN inhibition by bpV(pic)					
V_{57}	k_{57}	100	estimation		
	$K_{d,57}$	10	estimation IC_{50}=31 nM (4)		
**PI3K inhibition by LY294002**					
V_{58}	k_{58}	100	estimation		
	$K_{d,58}$	80	Estimation, K_i=1600 nM (5)		

[1)] the value of the parameters after rescaling of HRG and 2C4 concentrations to extracellular volume (2).

Table S3. Initial concentrations of the metabolites in the model, nM

**Species**	**Values in the model**	**Remarks**	**Values in the model** (1)	**Values in the model** (2)
HER3	80	estimation	HER4=80	EGFR=100
HER2	100	estimation		
HRG	100	estimation	100	
Shc	100	estimation	100	150
GS	100	estimation	10	
RasGDP	120	estimation	120	
Raf	100	estimation	100	
E_{Raf}	7	estimation	7	
MEK	100	estimation	120	
ERK	100	estimation	1000	
PI3K	200	estimation	10	
AKT	100	estimation	10	
PP2A	10	estimation	11.4	
PI	300	estimation	800	
PTEN	50	estimation		

2. Analysis of the pAKT dose dependence for pertuzumab

To confirm a non-zero limit of sensitivity $S_{AKT,k31}(2C4,p)$ (Fig. 5) we calculated the dependence of ΔpAKT on pAKT: ΔpAKT(pAKT) (see Fig. S1). As seen, ΔpAKT(pAKT) is a linear function of pAKT at high pertuzumab concentrations (low pAKT signal). Therefore the ratio ΔpAKT/pAKT tends to a constant that determines the limiting value of the relative sensitivity, ΔpAKT/pAKT/(p/Δp) (Eq. 4) to be a nonzero constant close to 1. This calculation corresponds to the black dashed line in Fig. 5.

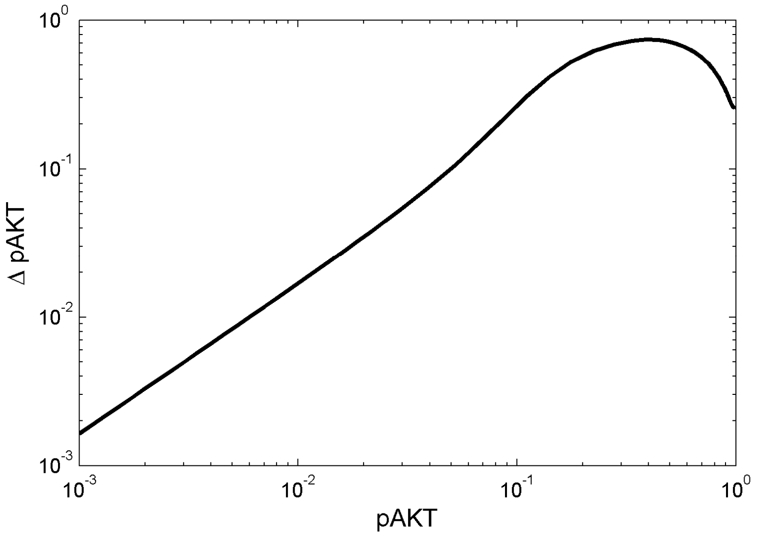

Fig. S1. The dependence of ΔpAKT on pAKT, ΔpAKT(pAKT) at high concentrations of pertuzumab (in double logarithmic scale) at a fixed relative change of parameter p, $\Delta p/p=10^{-2}$.

We compared relative and absolute sensitivity $S_{AKT,k31}(2C4,p)$ (see Figs. S2A and B, respectively). Figs. S2B shows the absolute sensitivities of pAKT with respect to the relative change of the parameters p (k_{31})

$$\mathbf{S}_{AKT,p}(2C4,p) = \left|\frac{\Delta pAKT}{\Delta p/p}\right|. \tag{S2.1}$$

As can be seen, the relative and absolute sensitivities have non-zero and zero limit values at high concentration of pertuzumab, respectively. The limit values of both sensitivities at low drug concentrations correspond to the sensitivities of the signalling system in the absence of drug (see heatmap for the relative sensitivity in Fig. 4).

Note, at 3 μM LY294002 absolute sensitivity (S2.1) monotonically decreases when the drug concentration increases and does not have maxima in the range of IC_{50} in contrast to that at low LY294002 concentrations.

A

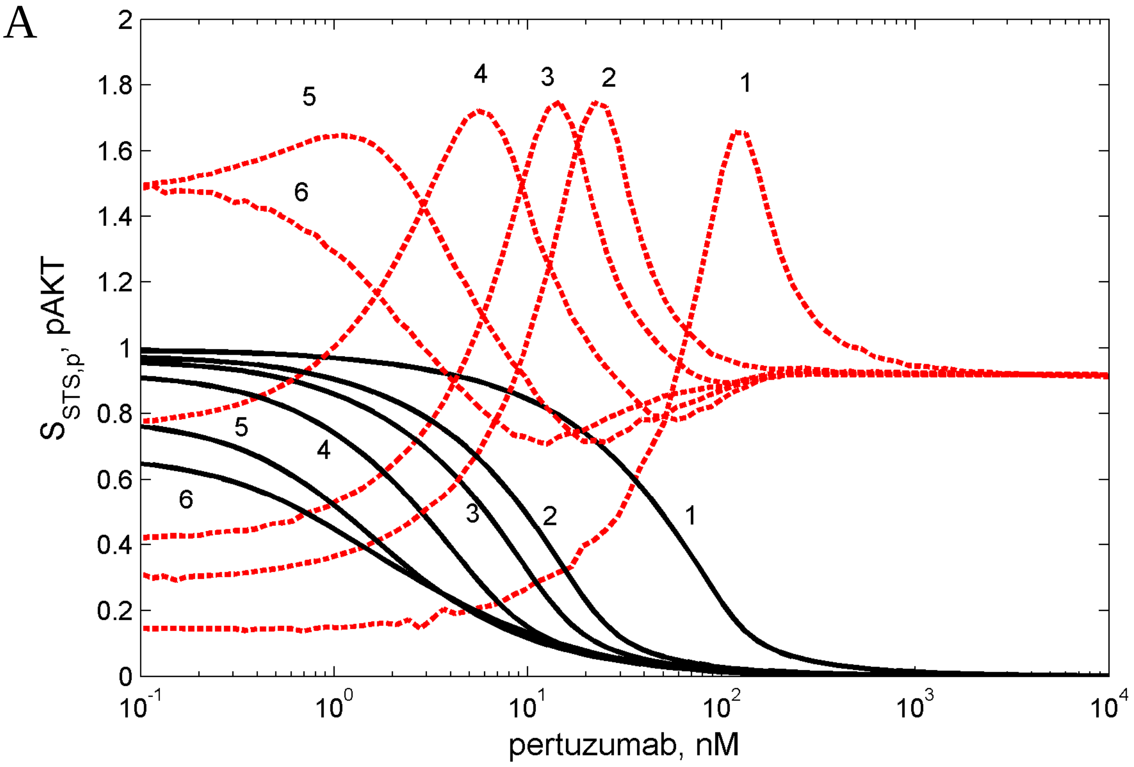

B

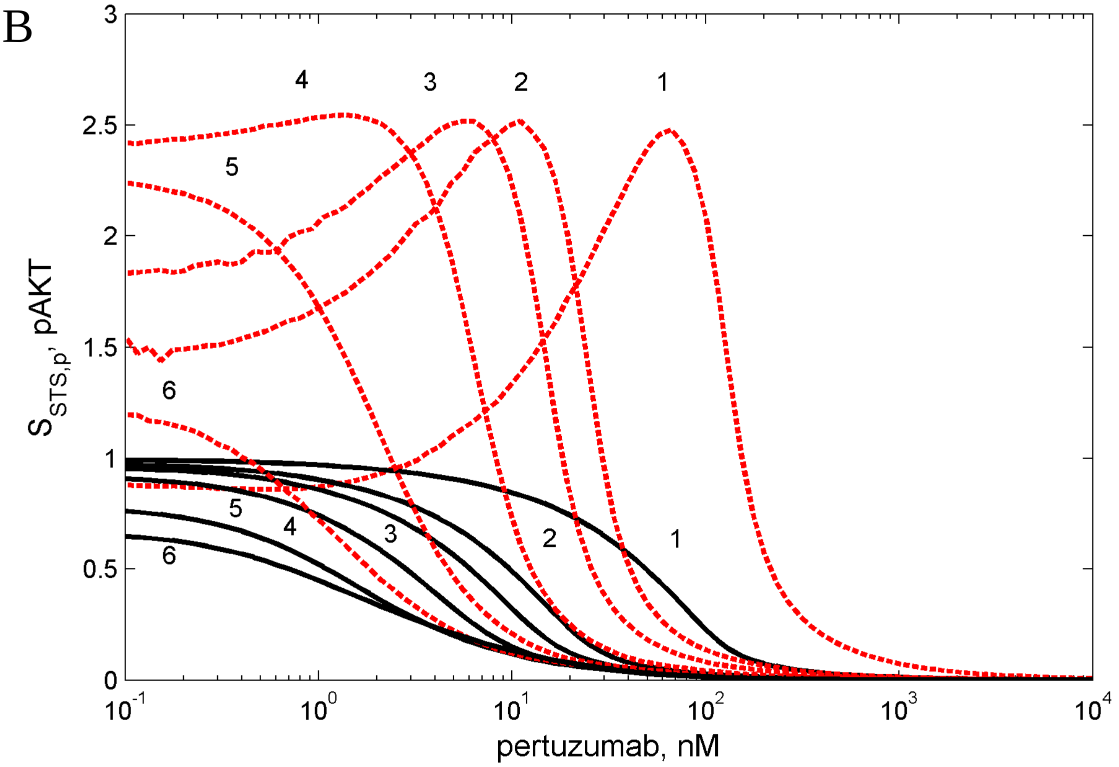

Fig. S2. The dose dependencies of pAKT inhibition (black solid lines) and relative (A) and absolute (B) sensitivity $S_{AKT,k31}(2C4,p)$ (red dashed lines) for pertuzumab (2C4) at the different concentrations of LY294002: 0 µM (lines 1); 0.3 µM (lines 2); 0.5 µM (lines 3); 1 µM (lines 4); 2 µM (lines 5); 3 µM (lines 6). A change in the dose dependence shape of the sensitivity is defined by the transformation of the pAKT response from graded hyperbolic to smooth sigmoid form (see Fig. 3A). Note the absolute sensitivity is divided by 15.

2.1. Analytical calculation of the limiting value of relative sensitivity $S_{AKT,p}(2C4,p)$ when HER2 inhibitor concentration tends to high concentrations

We carried out a numerical analysis of the limiting value of relative sensitivity $S_{AKT,k31}(2C4,p)$ as the concentration of HER2 inhibitor tends to high concentrations (see Figs. S1 and S2). The results of this analysis showed that the limiting value of $S_{AKT,k31}(2C4,p)$ is non-zero as the concentration of 2C4 tends to infinity. To confirm this result analytically we calculated the same limit for the Hill function which approximates the solution of ODEs (S1.1)-(S1.56) and describes the dose dependence of pAKT (black lines in Fig. S2):

$$Y(X) = \frac{K^n}{K^n + X^n}, \qquad \text{(S2.2)}$$

where X and Y are inhibitor concentration and pAKT signal value, respectively. K and n are the parameters of the Hill function which characterises internal properties of the signalling system. Using explicit approximating function (S2.2) we can calculate analytically relative sensitivity of Y to parameter K, $S_{Y,K}(X)$, and find its limit as X tends to infinity. $S_{Y,K}(X)$ has the form

$$S_{Y,K}(X) = \frac{\partial Y}{\partial K}\frac{K}{Y} = \frac{nX^n K^{n-1}}{\left(K^n + X^n\right)^2}\frac{K}{Y} = \frac{nX^n}{K^n + X^n}.$$

Then the limiting value is defined by

$$\lim_{X\to\infty} S_{Y,K}(X) = \lim_{X\to\infty} \frac{nX^n}{K^n + X^n} = n\,.$$

Thus, relative sensitivity Y to parameter K tends to non-zero limiting value as X tends to infinity that is in agreement with the numerical results (see Figs. S1 and S2). Note, that absolute sensitivity

$$\mathbf{s}_{Y,K} = \frac{\partial Y}{\partial K} = \frac{nX^n K^{n-1}}{\left(K^n + X^n\right)^2}$$

tends to zero as X tends to infinity in contrast to the relative sensitivity.

Though the Hill function of two parameters (S2.1) is a crude approximation of the exact solution of ODEs (S1.1)-(S1.56) depending on more than 50 parameters, the analytical results shows that the relative sensitivity $S_{AKT,p}(2C4,p)$ can have non zero limiting value as drug concentration tends to high concentrations.

References

1. Hatakeyama M, Kimura S, Naka T, Kawasaki T, Yumoto N, Ichikawa M, et al. A computational model on the modulation of mitogen-activated protein kinase (MAPK) and Akt pathways in heregulin-induced ErbB signalling. Biochem. J. 2003 Jul 15;373(Pt 2):451–63.

2. Kholodenko BN, Demin O V, Moehren G, Hoek JB. Quantification of short term signaling by the epidermal growth factor receptor. J. Biol. Chem. 1999 Oct 15;274(42):30169–81.

3. Adams CW, Allison DE, Flagella K, Presta L, Clarke J, Dybdal N, et al. Humanization of a recombinant monoclonal antibody to produce a therapeutic HER dimerization inhibitor, pertuzumab. Cancer Immunol. Immunother. 2006 Jun;55(6):717–27.

4. Schmid AC, Byrne RD, Vilar R, Woscholski R. Bisperoxovanadium compounds are potent PTEN inhibitors. FEBS Lett. 2004 May 21;566(1-3):35–8.

5. Vlahos CJ, Matter WF, Hui KY, Brown RF. A specific inhibitor of phosphatidylinositol 3-kinase, 2-(4-morpholinyl)-8-phenyl-4H-1-benzopyran-4-one (LY294002). J. Biol. Chem. 1994 Feb 18;269(7):5241–8.
